# CD81-guided heterologous EVs present heterogeneous interactions with breast cancer cells

**DOI:** 10.1186/s12929-024-01084-9

**Published:** 2024-10-15

**Authors:** Elena Gurrieri, Giulia Carradori, Michela Roccuzzo, Michael Pancher, Daniele Peroni, Romina Belli, Caterina Trevisan, Michela Notarangelo, Wen-Qiu Huang, Agata S. A. Carreira, Alessandro Quattrone, Guido Jenster, Timo L. M. Ten Hagen, Vito Giuseppe D’Agostino

**Affiliations:** 1 Laboratory of Biotechnology and Nanomedicine, Department of Cellular, Computational and Integrative Biology (CIBIO), University of Trento, Via Sommarive 9, 38123 Trento, Italy; 2 Advanced Imaging Core Facility, Department of Cellular, Computational and Integrative Biology (CIBIO), University of Trento, Via Sommarive 9, 38123 Trento, Italy; 3 High Throughput Screening and High Content Analysis Core Facility, Department of Cellular, Computational and Integrative Biology (CIBIO), University of Trento, Via Sommarive 9, 38123 Trento, Italy; 4 Mass Spectrometry and Proteomics Core Facility, Department of Cellular, Computational and Integrative Biology (CIBIO), University of Trento, Via Sommarive 9, 38123 Trento, Italy; 5 Precision Medicine in Oncology (PrMiO), Department of Pathology, and Nanomedicine Innovation Center Erasmus (NICE), Erasmus MC Cancer Institute, 3015 GD Rotterdam, The Netherlands; 6 Laboratory of Genomic Screening, Department of Cellular, Computational and Integrative Biology (CIBIO), University of Trento, Via Sommarive 9, 38123 Trento, Italy; 7 Laboratory of Translational Genomics, Department of Cellular, Computational and Integrative Biology (CIBIO), University of Trento, Via Sommarive 9, 38123 Trento, Italy; 8 Department of Urology, Erasmus University Medical Center, Dr. Molewaterplein 40, 3015 GD Rotterdam, The Netherlands

**Keywords:** Tetraspanin, Extracellular vesicles, Nanovehicles, Receptor-binding, Drug-loading, RNA cargo

## Abstract

**Background:**

Extracellular vesicles (EVs) are cell-secreted particles conceived as natural vehicles for intercellular communication. The capacity to entrap heterogeneous molecular cargoes and target specific cell populations through EV functionalization promises advancements in biomedical applications. However, the efficiency of the obtained EVs, the contribution of cell-exposed receptors to EV interactions, and the predictability of functional cargo release with potential sharing of high molecular weight recombinant mRNAs are crucial for advancing heterologous EVs in targeted therapy applications.

**Methods:**

In this work, we selected the popular EV marker CD81 as a transmembrane guide for fusion proteins with a C-terminal GFP reporter encompassing or not Trastuzumab light chains targeting the HER2 receptor. We performed high-content imaging analyses to track EV-cell interactions, including isogenic breast cancer cells with manipulated HER2 expression. We validated the functional cargo delivery of recombinant EVs carrying doxorubicin upon EV-donor cell treatment. Then, we performed an in vivo study using JIMT-1 cells commonly used as HER2-refractory, trastuzumab-resistant model to detect a more than 2000 nt length recombinant mRNA in engrafted tumors.

**Results:**

Fusion proteins participated in vesicular trafficking dynamics and accumulated on secreted EVs according to their expression levels in HEK293T cells. Despite the presence of GFP, secreted EV populations retained a HER2 receptor-binding capacity and were used to track EV-cell interactions. In time-frames where the global EV distribution did not change between HER2-positive (SK-BR-3) or -negative (MDA-MB-231) breast cancer cell lines, the HER2 exposure in isogenic cells remarkably affected the tropism of heterologous EVs, demonstrating the specificity of antiHER2 EVs representing about 20% of secreted bulk vesicles. The specific interaction strongly correlated with improved cell-killing activity of doxorubicin-EVs in MDA-MB-231 ectopically expressing HER2 and reduced toxicity in SK-BR-3 with a knocked-out HER2 receptor, overcoming the effects of the free drug. Interestingly, the fusion protein-corresponding transcripts present as full-length mRNAs in recombinant EVs could reach orthotopic breast tumors in JIMT-1-xenografted mice, improving our sensitivity in detecting penetrant cargoes in tissue biopsies.

**Conclusions:**

This study highlights the quantitative aspects underlying the creation of a platform for secreted heterologous EVs and shows the limits of single receptor-ligand interactions behind EV-cell engagement mechanisms, which now become the pivotal step to predict functional tropism and design new generations of EV-based nanovehicles.

**Supplementary Information:**

The online version contains supplementary material available at 10.1186/s12929-024-01084-9.

## Background

Extracellular vesicles (EVs) are cell-secreted lipid particles recognized as natural vehicles for intercellular communication [1, 2]. EVs derive from different biogenesis pathways and comprise exosomes, ectosomes, and apoptotic bodies. These particles differ from other nanometer-scale non-vesicular structures called exomeres and supermeres [3, 4]. Exosomes are small vesicles, typically ranging from 30 to 150 nm in diameter, formed within multivesicular bodies (MVBs). These particles are released in the extracellular space upon fusion of the MVB with the plasma membrane [5]. Endosomal sorting complex required for transport (ESCRT)-dependent or -independent mechanisms are either involved in the formation of MVBs and intraluminal vesicles, including proteins such as ALIX (ALG2-interacting protein X), TSG101 (tumor susceptibility gene 101) or SYNTENIN [6]. Ectosomes are typically larger EVs ranging from 150 nm to 1 μm. These vesicles are generated by direct outward budding of the plasma membrane in response to signaling and membrane rearrangements not yet clarified, including changes in lipid-protein composition and calcium-dependent mechanisms shaping the membrane symmetry in connection with the cytoskeleton [5]. Tetraspanins like CD81, CD63, and CD9 are widely detected on heterogeneous EVs and are mainly described in the ESCRT-independent mechanisms, forming clusters with transmembrane/cytosolic proteins involved in membrane budding [7]. On the other hand, apoptotic bodies are formed during the programmed cell death process [5].

The capacity to entrap heterogeneous molecular cargoes inside biocompatible and stable lipid particles [8] renders EVs an attractive source for developing targeted therapy formulations [9]. Indeed, EVs can exert anti-inflammatory and antitumor effects or neuroprotection and tissue regeneration [10–12] by delivering specific siRNAs, small molecules, microRNAs, or proteins [13, 14]. For these reasons, the latest research efforts focus on exploiting EVs for molecular diagnostics to identify prognostic or predictive biomarkers and, in parallel, developing formulations for targeted delivery applications [15].

Speculating on EV-cell engagement mechanisms by receptor-ligand interactions, the EV membrane could be conceived as a functionalization-prone surface that could orient a specific cargo delivery to desired cell populations. In the context of targeted delivery studies, the Human Epidermal Growth Factor Receptor 2 (HER2/ERBB2) exposed on selected tumor cells [16] represents a widely recognized model receptor. Aberrant protein expression and mutations in the *ERBB2* gene are found in different solid tumors, including breast cancer [17]. The monoclonal antibody Trastuzumab was the first anti-HER2 agent approved in combination with conventional chemotherapy for patients with HER2-positive metastatic breast cancer and subsequently approved as adjuvant therapy in patients with early-stage disease [18]. Therefore, taking advantage of fusion proteins containing ligands for the HER2 extracellular domain [19], EVs were engineered to expose these moieties and gain preferential distribution to HER2-positive tumor cells in vitro and in vivo, indeed serving as new tools for selective tumor imaging [20], delivering cytotoxic drugs [21, 22], or co-activating immune cells to suppress tumor growth [23–25]. To obtain functionalized EVs, cell-secreted particles already in suspension were directly manipulated through incubation mixtures with anti-HER2 moieties anchored in micelles and liposomes, with or without enzymatic ligation steps. These approaches are generally referred to as *post-isolation* strategies [26]. Conversely, *pre-isolation* strategies are based on manipulating EV-donor cells to express transmembrane or membrane-associated proteins with fusion ligands expected to be part of the EV surface. In this case, cells constitute a versatile platform for continuous secretion of functionalized EVs that can reach different tissues with desired ligand exposure and molecular cargo [26]. In particular, HEK293T cells were used to over-express a LAMP2b protein fused to a designed ankyrin repeat peptide detectable on secreted EVs, in turn capable of targeting HER2 in receptor-positive cells and inducing cytotoxicity through a doxorubicin cargo [22, 27]. The same cells were used to ectopically express anti-HER2 single-chain variable fragments (scFv) fused to the C1-C2 domains of lactadherin, generating EVs able to recognize the HER2 receptor and deliver prodrugs active in recipient cells [28, 29]. An alternative strategy was also based on the transmembrane domain of platelet-derived growth factor receptor (PDGFR) to express a CD3/HER2 bi-specific fusion protein for an EV-mediated T cell-killing activity in HER2-positive breast cancer cells [23]. These studies demonstrated the promising clinical utility of EVs secreted by other cell types (“heterologous EVs”) as viable cell-targeting formulations. However, we still lack quantitative profiling that could provide information on the density and quality of functionalized EVs over the bulk secretome, the potential contribution of cell-exposed receptors to EV distribution, and the predictability of functional cargo release. In addition, novel opportunities for heterologous EVs to package and share high molecular weight recombinant mRNAs need to be explored, prospecting comparative studies with other lipid nanoparticles and hybrid formulations to advance our portfolio of targeted delivery systems.

In this study, we selected a popular EV transmembrane protein to express fusion moieties in HEK293T cells and subsequently quantify the fraction of functionalized EV populations. Several studies already demonstrated the utility of recombinant tetraspanins, such as CD63, for capturing or tracking cell-secreted EVs in vitro and in vivo [30–32]. Indeed, CD81, CD9, and CD63 proteins can be detected in nearly all tissues [33] and currently represent hallmarks of cell-secreted EVs [34, 35]. Recent studies profiling single EVs indicated that CD81 was one the most efficient candidates for EV protein cargo loading [36] and presented higher levels in individual blood-circulating or mesenchymal stem cell-derived EVs compared to CD63 [37, 38]. Moreover, the fewer transcript variants of *CD81* (Gene ID: 975) compared to *CD63* (Gene ID: 967) and the higher structural homology with the other EV-accumulating CD9 protein [39] defined CD81 as an attractive guide for fusion proteins. For the first time, using the HER2 as a target receptor, we challenged HEK293T cells to induce secretion of EV populations with a CD81 protein fused to GFP with or without Trastuzumab light chains. After profiling cell-secreted particles and GFP-positive fractions in bulk EV populations, we found a strong correlation between intracellular expression levels of CD81-guided fusion proteins and the abundance of EVs containing the protein of interest. Moreover, we found that almost the total fraction of recombinant EVs, representing ~ 20% of secreted bulk EVs, retained anti-HER2 binding performance in vitro specifically conferred by the fusion ligand and predicting a receptor-competent binding configuration. We investigated the cell-targeting abilities using SK-BR-3 and MDA-MB-231 breast cancer cell lines exquisitely expressing or not, respectively, the HER2 receptor. Interestingly, we also tested isogenic cells that we established by inverting the dosage of HER2 receptor in the two parental cell lines, i.e., using CRISPR-Cas9-mediated knock-out in SK-BR-3 or HER2 over-expression in MDA-MB-231 cells. Through automated confocal imaging analyses using equivalent GFP-positive EVs, we show for the first time a remarkably affected tropism of antiHER2 EVs in the isogenic cell lines manipulated for HER2 expression. These data acquired more relevance in a context where the number of spots of antiHER2 EVs did not significantly differ from control EVs when comparing parental SK-BR-3 with MDA-MB-231 cells, except for a detectable increase in fluorescence intensity and spot areas in the HER2-positive SK-BR-3 cells, pointing out the spot-detection as an insightful readout to measure EV tropism in vitro. We functionally validated these findings by performing viability assays with recombinant doxorubicin-EVs, showing that cargo delivery of heterologous EVs can be predicted and quantified in vitro. As informed by the cumulative fraction of recombinant control EVs reaching the different cells and releasing bioactive drugs, our results suggested that EV engagement is not restricted to single receptor-ligand interactions, whose quantitative tracking become pivotal to optimizing the functionalization of heterologous EVs. Finally, we performed an in vivo study to detect horizontally exchanged recombinant RNAs, showing that heterologous EVs can share a more than 2000 nt-length recombinant mRNA in HER2-refractory, trastuzumab-resistant JIMT-1-engrafted tumors, providing novel perspectives for ultrasensitive detection of penetrant RNA cargo and comparative studies to advance heterologous EVs in clinical applications.

## Methods

### Plasmids and cell lines

Trastuzumab light chains 1 and 2 were obtained by Tebubio Srl and cloned in the CD81-GFP vector (OriGene, 7268 bp), obtaining the antiHER2 construct (CD81-antiHER2-GFP, 7901 bp) still fused to the turbo GFP reporter. Human embryonic kidney HEK293T (ATCC, CRL-3216), human breast cancer MDA-MB-231 (ATCC, HTB-26) and SK-BR-3 (AMSBIO, Abingdon, UK) cell lines were cultured under standard conditions in DMEM supplemented with 10% Fetal Bovine Serum, 2 mM L-Glutamine, and 100 U/ml penicillin–streptomycin (all Gibco). SK-BR-3 HER2-knockout (SK-BR-3 KO) were obtained using pSpCas9 BB-2A-Puro (PX459) V2.0 (Addgene, 9200 bp) containing a sgRNA sequence (5ʹ-TCATCGCTCACAACCAAGTG-3ʹ) targeting exon 7 of *ERBB2* (cloned by Twin Helix) and selected with puromycin (Sigma-Aldrich) for 4 days (1 μg/ml puromycin for 72 h, followed by 24 h at 2 μg/ml). MDA-MB-231 cells expressing HER2 (MDA-MB-231 HER2 OE) were obtained by transient transfection of the pCMV3-SP-N-HA vector (SinoBiological, 6086 bp) for 24 h. Cells were transfected using either Lipofectamine 3000 (Invitrogen) or polyethylenimine (PEI, Sigma Aldrich) according to manufacturer’s protocols.

### Cell fractionation and immunoblotting

Cell fractionation experiments were performed as already described [40] using lysis buffer (50 mM HEPES pH 8, 10 mM NaCl, 10 mM MgCl_2_, 1 mM DTT, 10% glycerol, 1X protease inhibitor cocktail) supplemented by 25 µg/ml Digitonin (buffer A), 1% Igepal (buffer B) or 1% Triton X-100 and 1% Sodium deoxycholate (buffer C) for the sequential incubation and centrifugation protocol. Input samples corresponded to 2% of whole cell lysate from all the conditions analyzed. The first supernatant (cytosolic fraction) was collected after incubation of cells with buffer A on a rotary shaker for 10 min at 4 °C, then centrifuged at 2,000 rcf for 10 min at 4 °C. The obtained pellet was resuspended in ice-cold buffer B and vortexed before incubation on ice for 30 min and centrifuged at 7000 rcf for 10 min at 4 °C. The resulting supernatant corresponded to the organelle-enriched fraction, while the pellet was resuspended in ice-cold buffer C with the addition of benzonase (Novagen) and incubated on a rotary shaker for 30 min at 4 °C. Next, samples were sonicated at 4 °C for 45 s at 35 Amplitude (three cycles of 10 s on and 5 s off) within a ultrasonic bath sonicator (Q700, QSonica) and centrifuged at 7,800 rcf for 10 min at 4 °C to collect the nuclear fraction. Each fraction was loaded on 13% polyacrylamide gel for SDS-PAGE.

Protein concentration was measured in triplicate with the bicinchoninic acid (BCA) protein assay kit (Thermo Fisher Scientific). Immunoblotting experiments were performed as already described [41] using the following antibodies: tGFP (TA150041, OriGene), SYNTENIN (ab133267, Abcam), CD9 (ab236630, Abcam), CALNEXIN (ab22595, Abcam), HER2 (ab237715, Abcam), RAB5 (C8B1, 3547, Cell Signaling Technology), GAPDH (GTX627408, GeneTex), H3 (GTX122148, GeneTex), SERCA2 (ab2861, Abcam), TSG101 (GTX70255, GeneTex), secondary antibodies Peroxidase AffiniPure Goat Anti-Mouse and Anti-Rabbit IgG (H + L) (Jackson ImmunoResearch).

### EV isolation and characterization

For EV collection, HEK293T were plated in 100 mm TC-treated Culture Dish (Corning) and transfected with 3 µg plasmid/dish when at 75% of confluence. Forty-eight hours later, cells were washed with PBS and incubated for additional 24 h with serum-free medium. Media were centrifuged at 2,800 rcf for 15 min to eliminate cell debris and bigger particles. The supernatant was transferred to ultracentrifuge tube (38.5 ml-Beckman Coulter Ultra-Clear™) for ultracentrifugation at 100,000 rcf for 70 min at 4 °C using a Optima XE-90 ultracentrifuge with a SW 32 Ti rotor (Beckman Coulter). The EV pellet was resuspended in 0.22 μm-filtered sterile PBS and freshly-used or stored at -80 °C until further use. Nanoparticle tracking analysis (NTA) was performed using a NanoSight NS300 instrument (Malvern Panalytical, Malvern, UK) with a 532 nm laser. Each sample was subjected to 3–5 consecutive 60 s videos recorded at camera level 15. Particle concentration and size distribution were determined using NanoSight NS300 software NTA 3.4 Build 3.4.003 (Malvern Panalytical), setting 4 as a detection threshold.

Imaging flow cytometry was performed using an ImageStreamx (ISX) MKII instrument (Luminex Corporation) at 60 × magnification, high gain mode, and low flow rate. EV samples in PBS were labeled with 1 μg/ml (final concentration) of Cell Mask Deep Red (CMDR, Invitrogen) in ratio 1:1 (v/v) and incubated at RT for 20 min. Then, samples were diluted in PBS to obtain a final concentration lower than 10^10 objects/ml before acquisition. All samples were analyzed using INSPIRE® software (Luminex Corporation, Seattle, WA, USA), with a minimum of 3,000 events collected. Data analyses were performed using ISx Data Exploration and Analysis Software (IDEAS®, Luminex Corporation, Seattle, WA, USA).

Cryogenic electron microscopy (Cryo-EM) acquisitions were performed at Fisher Scientific (Eindhoven, The Netherlands). QuantiFoil 1.2/1.3 cu 200 grids, pretreated with 30 s glow discharging, were used. Vitrobot parameters were set as follows: 3.0 μl of each sample, temperature 4 °C, humidity 95%, blot time 7 s, blot force 0. Fifteen cryo-electron images were collected per each sample using a transmission electron microscope Glacios (Thermo Fisher Scientific), equipped with a Falcon 4i Selectris camera, at a nominal magnification of 49,000x. Imaging parameters were set as follows: Pixel size (Å) 2.4, Dose rate (e/pix/sec) 12.4, Total dose (e/Å^2) 20, Exposure time (sec) 9.3, Energy filter (eV) 10, defocus -1.9 μm. For image analysis, vesicular structures were manually selected as regions of interest (ROIs) using FIJI software and the size measured in pixels. From the ROI's pixel size, the actual area and diameter of EVs were calculated.

### AlphaLISA interaction assay

The Amplified Luminescent Proximity Homogeneous Assay (ALPHA Assay) was performed in white 384-well Optiplates (PerkinElmer) in a final volume of 20 μl. The antiHER2 protein was produced from CD81-antiHER2-tGFP vector (600 ng) using TNT® Quick Coupled Transcription/Translation Systems (Promega), following manufacturer’s instructions. The resulting product was pre-incubated with 30 nM HER2-DDK (TP322909, OriGene) for 30 min before addition to the other components: 10 nM tGFP Ab (TA150041, OriGene), anti-FLAG donor and protein G coated acceptor beads (10 ng/μl final concentration; AS103D and AL102C, PerkinElmer). For the competitive assay with CD81-GFP and antiHER2 EVs, 40 nM HER2-DDK and 9 nM anti-HER2 Ab (ab237715, Abcam) were used. All the components were diluted in the Alpha Screen Control Buffer (Perkin Elmer). The signal was detected using an EnSight® multimode plate reader (PerkinElmer) after 1 h of plate incubation at RT in the dark under rotation at 70 rpm.

### Immunoprecipitation and immunofluorescence

Serum-free media from transfected HEK293T was concentrated to about 2 ml with Amicon Ultra-15 Centrifugal Filter 10 kDa MWCO (Merck Millipore) at 3,000 rcf for 15–20 min, and diluted according to the relative concentration of GFP-positive particles. Input samples were prepared from 15 μl of undiluted conditioned medium. Anti-FLAG M2 magnetic beads (Sigma-Aldrich) or protein G dynabeads (Invitrogen) were washed three times in PBS before pre-incubation, at 4 °C for 1.5 h under rotation, with HER2-DDK or tGFP antibody, respectively. Bead-antibody mixtures were added to the media for 20–30 min at 4 °C under gentle rotation. For competitive HER2 binding, 1 μg/sample of Trastuzumab (anti-HER2-Tra-hIgG1, InvivoGen) was added directly to the media before incubation with beads. After the final incubation, beads were washed with PBS and resuspended with 1X Laemmli sample buffer for subsequent denaturation at 98 °C for 5 min and SDS-PAGE.

Immunofluorescence for HER2 detection was performed in breast cancer cell lines fixed with 4% paraformaldehyde for 15 min at RT and washed three times with cold PBS. Blocking (10% FBS, 0.05% Triton X-100 in PBS) was performed for 1 h at RT followed by primary Ab (ab237715, Abcam, diluted 1:1000, 0.1% FBS in PBS) incubation for 1 h at RT. After three washes with PBS, cells were incubated for 1 h at RT with Goat anti-Rabbit Alexa Fluor™ 633 (Invitrogen, diluted 1:1000, 0.1% FBS in PBS). Three washes with cold PBS 5 min each were performed before the addition of Hoechst for 15 min. One final wash with cold PBS was performed before acquisition. For CD81 and RAB5 IF, HEK293T were seeded on optical coverslips and IF was similarly performed, with the addition of a 5 min permeabilization step (0.1% Triton X-100 in PBS) before primary Ab incubation overnight at 4 °C (0.1% FBS and 0.05% Triton X-100 in PBS). CD81 Ab (MA5-13,548, Invitrogen) or RAB5 Ab (C8B1, 3547, Cell Signaling Technology). Goat anti-Mouse or anti-Rabbit IgG (H + L) Cross-Adsorbed Secondary Antibody Alexa Fluor™ 568 (Invitrogen) were used for the assays. Coverslips were mounted using ProLong Diamond Antifade Mountant (Invitrogen).

### Confocal microscopy and image analysis

Time-lapses and images of EV uptake by recipient breast cancer cells were acquired at the Optical Imaging Centre (OIC) at Erasmus MC (Rotterdam, The Netherlands) with a LEICA TCS SP8 AOBS confocal microscope, with Galvo Z stage and Adaptive Focus Control, using a HC Plan Apo CS2 40x/1.3 oil immersion objective. Live cell confocal imaging was performed under humidified conditions with 5% CO2 at 37 °C. MDA-MB-231 and SK-BR-3 cells were seeded in glass-bottom dishes (CELLview™ Culture dish, 35 mm, four chambers) and, before acquisition, 340 μl DMEM already containing 1.25 nM LysoTracker™ Red DND-99 (L7528, Invitrogen) and 1 µg/ml Hoechst (62,249, Thermo Scientific) were added to each chamber for 15 min. Before starting acquisition CD81-GFP or antiHER2 EVs were added in a ratio of 20,000–50,000 per seeded cell, in 350 μl as final volume. For fixed cell acquisitions, MDA-MB-231 (WT and HER2 OE) and SK-BR-3 (WT and KO) were seeded in the same dishes and incubated with EVs for 4 h considering the relative abundance of GFP-positive EVs, then washed with PBS before fixation and immunofluorescence (IF) for HER2 receptor. Fourteen Z-stacks were acquired within around 11 μm of total Z size, with voxel size 0.1623 × 0.1623x0.7991 μm^3^. Images were processed and analyzed using FIJI/ImageJ software.

To quantify EV-cell interactions, an automated pipeline was applied using CellProfilerTM version 4.0.7 on the Maximum Intensity Projection of 14 z-stacks for each acquired channel. Cell nuclei were identified as primary objects with a threshold in the Hoechst blue channel, the cytoplasms were defined as secondary objects using a low threshold in the HER2 red channel that allowed the segmentation of a cytoplasmic region also in the HER2 negative cells. EVs were defined as green spots in a range of diameters from 0.8 to 5 µm and the interaction within the cells has been established overlapping the EVs object mask to the cytoplasmic region using the object processing function “RelateObject”. Objects number, AreaShape and Red Intensity features were calculated for the identified Nuclei, Cytoplasms and EVs. The final Spreadsheets have been combined and the dataset has been analyzed using KNIME Analytics Platform v 4.7.5.

**Fig. 1:**
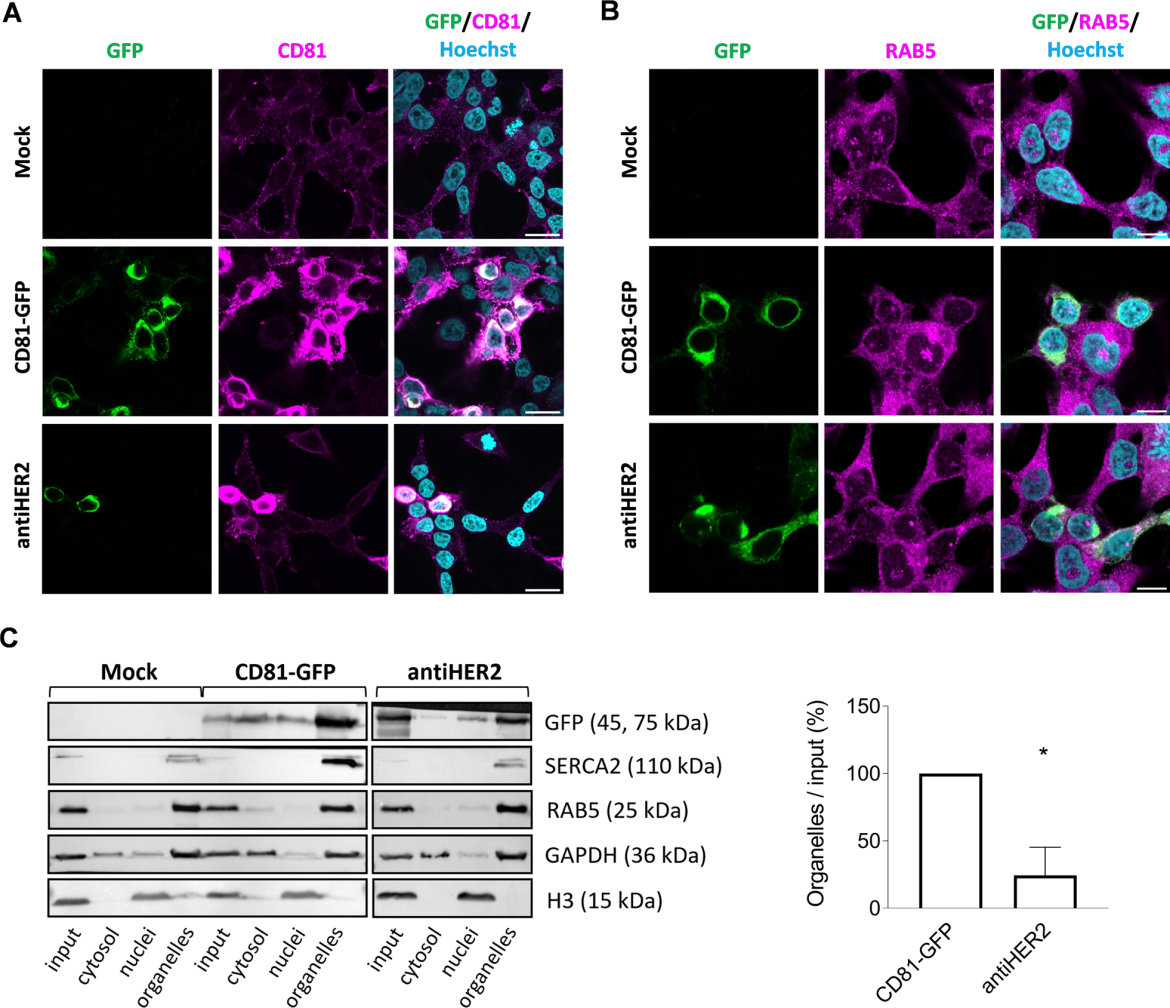
CD81 fusion proteins are expressed in HEK293T upon transient transfection and co-sediment with organelle-enriched sub-cellular fractions. **A, B** GFP detection and immunofluorescence staining of endogenous CD81 and RAB5 proteins in transfected HEK293T cells. Cell were subjected to confocal microscopy after 48 h of transfection with CD81-GFP and antiHER2 plasmids. Recombinant proteins are visualized in green (GFP), endogenous CD81 or RAB5 in magenta (Alexa Fluor 568), and cell nuclei in cyan (Hoechst). Scale bar is 20 μm in A and 10 μm in B. **C** Immunoblotting of sub-cellular fractions obtained through a sequential lysis buffer-centrifugation protocol. Separation of subcellular fractions was confirmed by the enrichment of corresponding protein markers: Cytosol (GAPDH), nuclei (histone H3), and organelles (SERCA2 for endoplasmic reticulum, RAB5 for early-endosomes). GFP-positive chimeric proteins were detected at the expected molecular weight (45 for CD81-GFP and 75 kDa for antiHER2). The histogram reports the densitometric quantification normalized over CD81-GFP condition, with mean and SD of two independent experiments. Significance is *P < 0.05

Images in Fig. 1A, B were acquired with a Nikon AX laser scanning inverted confocal microscope, using a Plan Apo H 60x/1.4 oil immersion objective. Images in Fig. 4A and Fig. S3B were acquired with a Nikon Ti2 inverted microscope equipped with a Crest X-light V2 Spinning Disc system and an Andor iXon Ultra 888 EMCCD camera, using a Plan Apo 20x/0.75 objective. Images in Fig. S1B were acquired with a Nikon Ti2 inverted microscope equipped with a Crest X-light V2 Spinning Disc system and an Andor Zyla 4.2 PLUS sCMOS camera, using a Plan Apo 20x/0.75 objective. For all the acquisitions, settings were kept constant within the same experiment and linear adjustments for brightness and contrast were equally applied to the reported images.

### RNA isolation and digital droplet PCR

Total EV-RNA was extracted with TRIzol reagent (Ambion, Life Technologies) and chloroform precipitation, followed by single-cell RNA purification (Norgen kit) including on-column DNAse I (Qiagen) treatment for 10 min at RT. cDNA synthesis was performed following manufacturer’s instructions (SensiFAST™, Meridian Bioscience™) starting from 15 μl of RNA template. To assess the integrity of the fusion protein-encoding transcripts (CD81-GFP, antiHER2), the corresponding cDNA samples were amplified prior DNA electrophoresis. ddPCR experiments were carried out using EvaGreen, following manufacturer’s instructions (Bio-Rad). EV-derived cDNA samples (5.5 μl each) were mixed with 11 μl of 2X QX200™ ddPCR™ EvaGreen Supermix (Bio-Rad) and 5.5 μl of primers (35 nM each). The following primers were used to specifically amplify the fusion sequences of interest: A) 5’-CTTCAAGGAGGACTGCCAC & 5’-GCTCGCGCTATAAATCAGCAGT; B) 5’-TGACCAAAAGCTTTAACCGTG & 5’-TGGGGTAGGTGCCGAAGT; C) 5’-CTTCAAGGAGGACTGCCAC & and 5’-TGGGGTAGGTGCCGAAGT) and target concentration was determined using QuantaSoft Software™ (Bio-Rad).

RNA extraction from tumor xenografts was performed with TRIzol and RNeasy Mini Kit (Qiagen), following the manufacturer’s instructions. cDNA synthesis was carried out using WarmStart reverse transcriptase (New England Biolabs) from 6 μg of RNA as starting material, including couple B primers (160 nM each). RNAse H from E. coli (Illumina) was added (10U/sample) for 30 min at 37 °C. cDNA purification was performed using the Nucleospin gel and PCR clean-up kit (Macherey–Nagel) following manufacturer’s instructions. ddPCR reactions were performed with cDNA diluted 1:300.

### UHPLC-MS analysis of secreted doxorubicin

To collect doxo-EVs from drug-treated cells, transfected HEK293T were incubated with 10 μM doxorubicin (BD32885, BLD Pharmatech GmbH) for 3 h at 37 °C and 5% CO2. After a PBS wash, EVs were isolated from serum-free DMEM as previously described. Metabolite extraction was carried out by adding 80% cold methanol to the EV stocks. Samples were then vigorously shaken (5 min) and kept at -80 °C overnight. Finally, the samples were vacuum-dried using a SpeedVac concentrator. Dried extracts were equilibrated to RT, resuspended in 30 µl of acetonitrile:water:formic acid (5:95:0.1%, v/v/v), and thoroughly mixed. Seven serial drug dilutions, ranging from 8,000 to 1.95 nM, were used to prepare the standards for the calibration curves. Ten μl of standards and samples were injected onto Ultimate 3000RS (Thermo Scientific) UHPLC system coupled online with an Orbitrap Fusion Tribrid (Thermo Scientific) mass spectrometer. A Hypersil Gold C18 column (Thermofisher, 100 × 2.1 mm, particle size: 1.9 µm) was used for separation. The LC method consisted of a linear gradient from 5 to 100% B (B: acetonitrile 0.1% of formic acid; A: water + 0.1% formic acid) over 15 min, followed by 4 min at 100% at the flow of 0.2 ml/min. The MS spray voltage was set at + 3500 V with the ion transfer tube temperature set at 300 °C (sheath and auxiliary gasses were set at 20 and 5 Arb, respectively). The MS data were acquired in full scan in the Orbitrap at 120.000 FWHM (200 m/z), in the scan range of 100–1000 m/z. Software FreeStyle ver.1.6 (Thermo Scientific) was used to inspect mass spectra. The area under the peak of each precursor ion and the total ion current (TIC) were extracted using Skyline (MacCoss Lab Software) [42]. The software derived precise m/z values as well as isotope distributions for each precursor. Each analyte was also investigated for common adducts, [M + H] + , [M + K] + , [M + NH4] + , [M + Na] + , and the [M − H2O + H] + ions, for each considering the three most abundant isotopes. The total area of precursor ions was calculated by summing all precursor levels. The log2-transformed value of the total area of precursor ions of each standard sample was plotted as function of the concentration to construct the standard curves of each compound.

### Cell viability assay

The 3-(4,5-dimethylthiazol-2-yl)-2,5-diphenyl-2H-tetrazolium bromide (MTT, Thermo Scientific) assay was performed on cells seeded and treated in 96 plates for 72 h with the free doxorubicin or doxo-EVs. Doxorubicin concentration in doxo-EVs was estimated from previous LC–MS analysis. After incubation, the medium was removed and MTT solution (0.5 mg/ml in DMEM) incubated for 4 h at 37 °C and 5% CO2, before cell lysis in DMSO. Absorbance (570 nm) was measured at a Varioskan LUX Multimode Microplate Reader (Thermo Fisher Scientific) and cell viability was calculated as % with respect to untreated cells (DMEM only).

### In vivo study

The in vivo study was performed in collaboration with Reaction Biology Europe GmbH (Freiburg, Germany). Each experimental groups contained five female athymic nude mice (Crl:NU(NCr)-Foxn1nu). On Day 0, 5.0 × 10^6^ JIMT-1 human breast carcinoma tumor cells in 100 µl PBS were implanted into the left mammary fat pad of each mouse. After animals had been randomized on Day 12, treatments of the test samples were initiated. All treatments were administered at a dose of 0.5 µg/kg and a dosing volume of 5 ml/kg subcutaneously at the tumor implantation site. Animal weights were measured three times, one time on day of randomization (Day 12) and two times after the start of therapy (Days 13 and 15). During the study, the growth of the intramammary implanted JIMT-1 primary tumors was determined twice by caliper measurement on Days 12 and 15. All animals reached the end of the study as scheduled. The primary tumor samples were collected during the final necropsy on Day 15, the study endpoint.

### Statistical analysis

Statistical analysis was performed using GraphPad Prism 9, as well as data visualization, applying non-parametric Anova Kruskal–Wallis or Student’s *t*-test. A minimum of 95% confidence level was considered significant. The significance level was set at *P < 0.05; **P < 0.01; ***: P < 0.001; ****P < 0.0001; ns: not statistically significant. Mean and standard deviation of independent experiments are reported in the graphs and detailed in figure legends. Schematics were created in biorender.com.

## Results

### CD81 fusion proteins participate in intracellular vesicular trafficking

Recombinant plasmids were designed to encode a human full-length CD81 protein in frame with the turbo GFP reporter (CD81-GFP) and including Trastuzumab light chains 1 and 2 (CD81-antiHER2-GFP, or antiHER2), generating two fusion proteins with an expected molecular weight of 45 and 75 kDa, respectively (Fig. S1A). After 48 h of HEK293T cell transfection, the CD81-GFP protein showed the highest fluorescence intensity, which was slightly reduced in the case of the antiHER2 fusion protein. In this setting, chimeric proteins populated the perinuclear region, including a partial granular cytoplasmic and plasma membrane distribution matching the endogenous CD81 protein, in contrast to the whole cell body stained by GFP alone (Fig. 1A and Fig. S1B). By counterstaining cells with an antibody recognizing RAB5, a marker of early endosomes [43, 44], we found consistent cytoplasmic areas overlapping with GFP in confocal microscopy (Fig. 1B). Subsequent cell fractionation and immunoblotting experiments (Fig. 1C) indicated a co-sedimentation of fusion proteins in RAB5/Sarco-Endoplasmic Reticulum Calcium ATPase (SERCA)-positive (organelle-enriched) fractions according to relative protein expression levels and confirming the immunofluorescence evidence. These data supported the potential involvement of ectopic tetraspanin-guided fusion proteins in vesicular trafficking without interference with HEK293T cell viability.

### CD81 fusion proteins can be detected on secreted EV populations

**Fig. 2:**
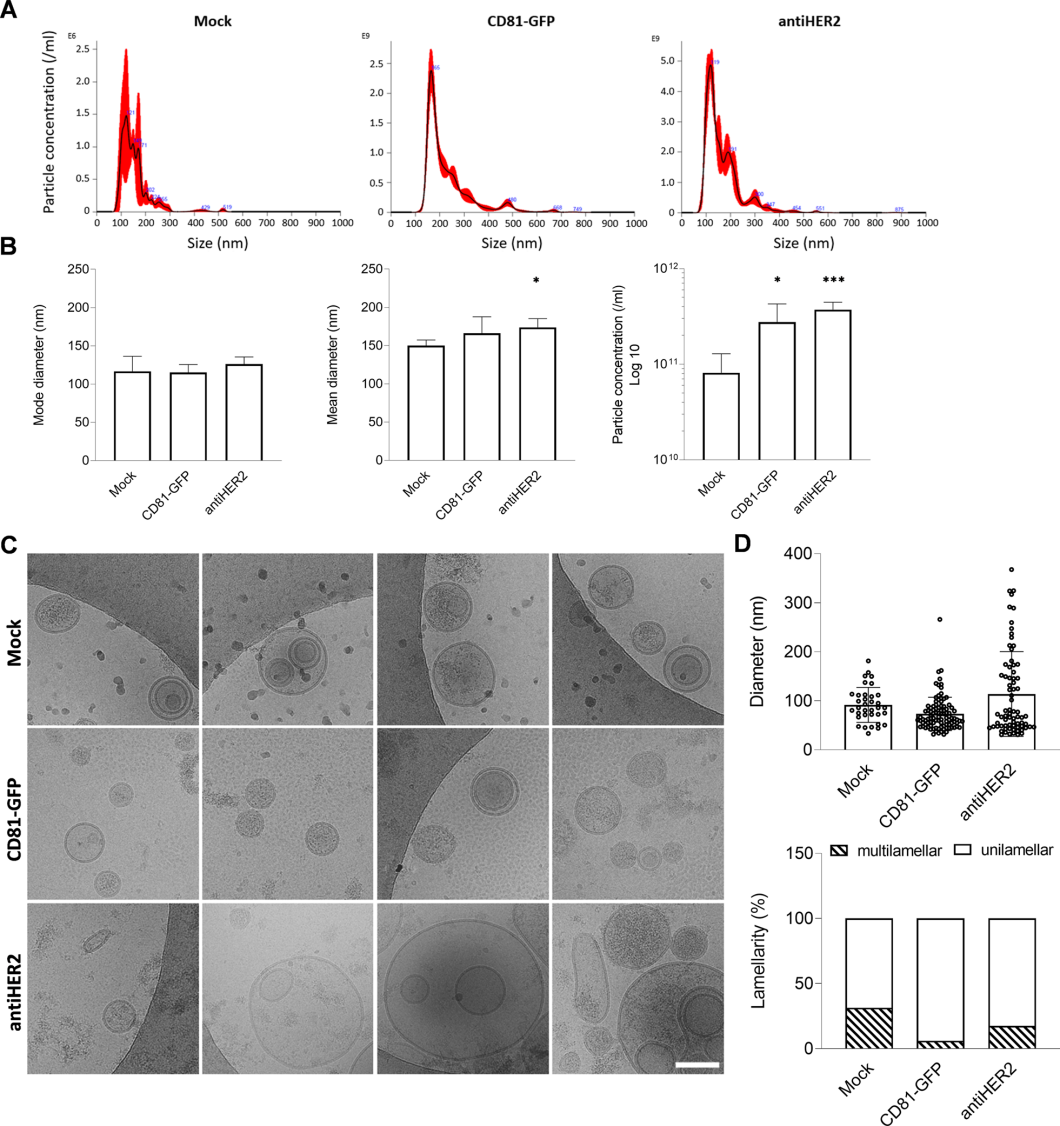
Profiling of particles recovered from transfected HEK293T cells. **A, B** Nanoparticle Tracking Analysis (NTA) of particles secreted by transfected HEK293T cells. Representative size distribution profiles of Mock, CD81-GFP, anti-HER2 samples. The black curve indicates the mean of three measurements, with SE in red. Mode and Mean diameters, and particle concentration are plotted. Error bars include at least three biological replicates. Significance * is P < 0.05, ***P < 0.001, *vs* Mock condition. **C** Representative Cryo-EM images of Mock, CD81-GFP and antiHER2 EV samples confirming the vesicular structure and size heterogeneity of recovered vesicles. The indicated scale bar is 100 nm. **D** Plot of the observed diameter of vesicles in Cryo-EM images (n = 35 for Mock, n = 99 for CD81-GFP, n = 74 for antiHER2) and lamellarity, expressed as percentage of unilamellar and multilamellar vesicles over the observed bulk EV populations

To investigate the presence of chimeric proteins in cell-secreted EVs, transfected HEK293T cells were exposed to serum-free media for 24 h. Media were subjected to differential ultracentrifugation (DUC), and recovered particles were characterized by nanoparticle tracking analysis (NTA), cryogenic electron microscopy (Cryo-EM), immunoblotting, and imaging flow cytometry. NTA experiments showed a heterogeneous particle size distribution in all the conditions analyzed, with the most represented particle populations between 100 and 200 nm (Fig. 2A). Although no significant changes were detected in mode diameters, the mean diameters showed a rising trend with ectopic CD81 proteins, contributing to a significant shift of about 15–25 nm in the case of antiHER2 particles (Fig. 2B). In parallel, the two ectopic proteins, especially antiHER2 (p value = 0.0005), were associated with a significant increase in abundance compared to Mock samples (Fig. 2B).

**Fig. 3:**
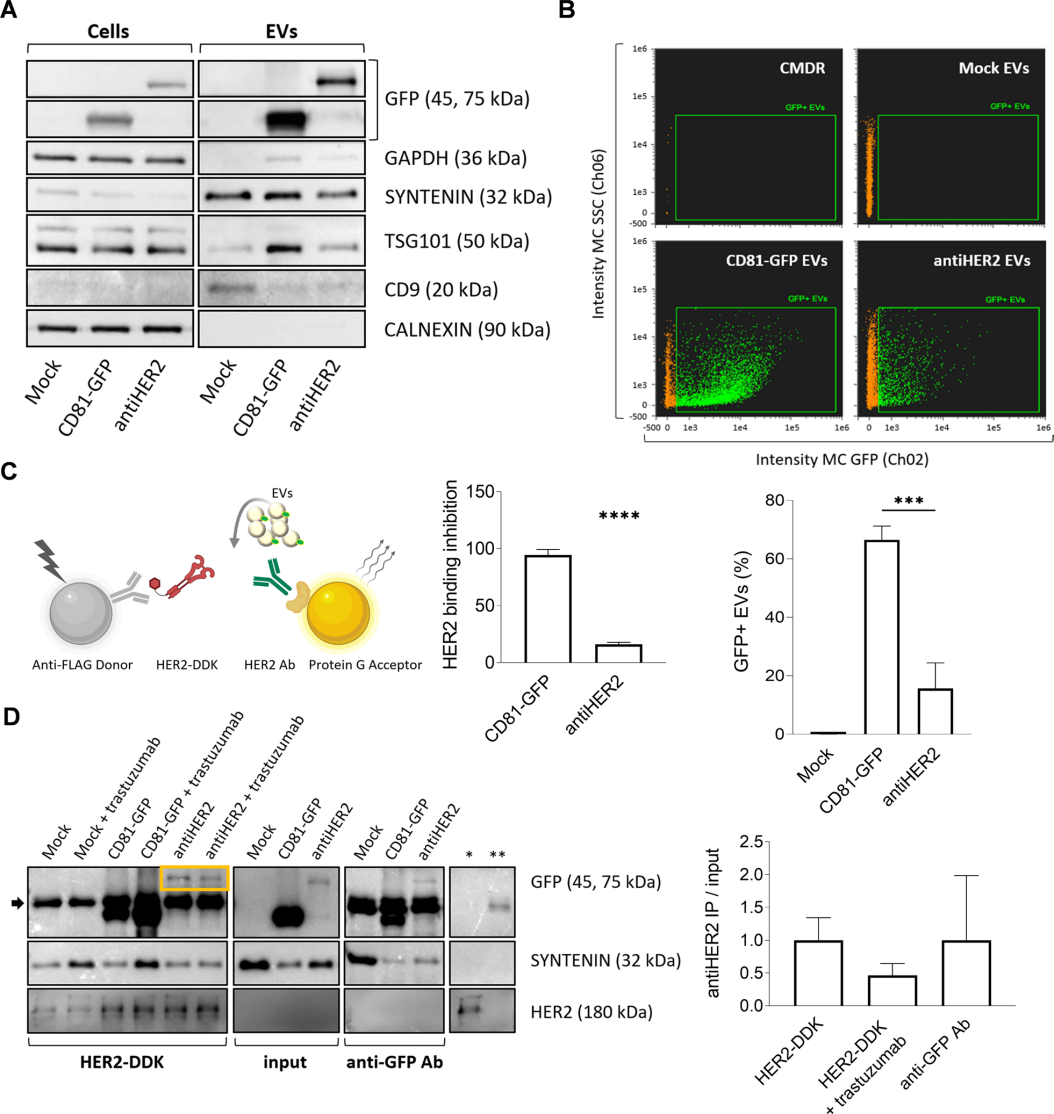
CD81-guided fusion proteins are cargo of secreted EV populations. **A** Representative immunoblotting of cell and EV lysates (1 μg proteins/well). EVs are positive to transmembrane (CD9) and cytosolic proteins (SYNTENIN, TSG101), while negative to CALNEXIN, and with low detectable levels of GAPDH compared to cell lysates.** B** Dot plots of imaging flow cytometry to detect GFP-positive EVs. The green fluorescent signal (Ch02, 488 nm laser) was detected as sub-gating of EVs labeled with Cell Mask Deep Red (CMDR, in orange, Ch11, 635 nm) to side-scatter (Ch06). Non-fluorescent, calibrator SpeedBeads, Amnis (1 µm) were continuously run during acquisitions. The graph shows the quantification of double-positive particles. Mean and error bars derive from three independent experiments. Significance antiHER2 vs CD81-GFP EVs is ***P < 0.001. **C** Sandwich designed for the AlphaLISA competitive assay. CD81-GFP and antiHER2 EVs were tested for competition with HER2-DDK. The graph shows the measured alpha counts normalized to the GFP-positive EV population as calculated by NTA and imaging flow cytometry. Mean and SD derive from three independent experiments (significance is ****P < 0.0001).** D** Representative western blotting of recombinant EVs immunoprecipitation with HER2-DDK or anti-GFP antibody in serum-free DMEM. AntiHER2 GFP-positive fusion proteins are enclosed in the yellow box above the antibody heavy chains (black arrow). Controls of beads flow through with HER2-DDK (*) or anti-GFP antibody (**) are shown on the right, indicating saturation of the beads’ surface to avoid non-specific binding. The graph shows the densitometric quantification of antiHER2 EVs captured by both HER2-DDK and anti-GFP Ab, with a competition effect of Trastuzumab. Mean and SD refer to two independent experiments

To gain insights into the quality of released particles, we acquired Cryo-EM images (Fig. 2C). Morphological analyses indicated an almost intact lipid bilayer with a predominant spherical shape for most retrieved particles, indeed confirmed as EVs. Notably, the image analysis revealed subsets of larger vesicles in the antiHER2 condition (Fig. 2D), mirroring the results obtained by NTA. In addition, Cryo-EM acquisitions systematically showed multi-layered (or multi-lamellar) vesicles, representing 10–30% of total EVs, whose fraction showed a trend of decrease in the ectopic conditions compared to Mock (Fig. 2D). Immunoblots on the same EV samples revealed the presence of chimeric proteins at the corresponding molecular weight, with concomitant accumulation of CD9 and SYNTENIN EV-positive markers, in contrast to the EV-negative marker CALNEXIN, which was undetected in EVs when compared to cell lysates (Fig. 3A). Further, to substantiate fusion proteins as part of EV protein cargo, we analyzed non-denatured samples by imaging flow cytometry using Cell Mask Deep Red (CMDR), a fluorescently-labeled dye with an affinity for lipid membranes. Once gated for CMDR positivity, the fraction of GFP-positive EVs was quantified (Fig. 3B). CD81-GFP EVs presented the highest percentage of double-positive events compared to antiHER2 EVs (66.5 ± 4.7% *vs* 15.65 ± 8.76%), with a consistent no signal in the Mock vesicles. Overall, CD81 fusion proteins were confirmed as cargoes of heterogeneous EV populations mirroring the relative recombinant protein abundance at the intracellular level.

### AntiHER2 fusion protein retains HER2 receptor binding and competition capacity in vitro

Since the mature endogenous CD81 protein is engaged in vesicular membranes [45], we investigated the exposure of CD81 fusion proteins on recovered EV populations. To this aim and further track EV-cell interactions, we designed biochemical assays to test the HER2-receptor binding of secreted EVs with fusion proteins retaining the GFP reporter. We developed an AlphaLISA assay using a human DDK (or FLAG)-tagged HER2 receptor recognized by anti-FLAG Donor beads and an in vitro-translated antiHER2 fusion protein produced by the CD81-antiHER2-GFP construct. The interaction was measured in the presence of a monoclonal anti-GFP antibody coupled with Protein G Acceptor beads. When all these ligands were present, we obtained a specific signal in the nanomolar range demonstrating the affinity of antiHER2-GFP fusion protein for the HER2 receptor (see schematics and results in Fig. S2A). The binding capability was also confirmed by detecting the HER2 receptor using a commercially available anti-HER2 antibody, confirming the utility of the assay to quantify the binding efficiency of the ectopically expressed antiHER2-GFP protein (Fig. S2B). We therefore exploited these settings using the optimal bead:ligand ratio (hooking point) [46, 47] to perform AlphaLISA competitive assays. We measured the binding efficiency of secreted antiHER2 EVs compared with secreted CD81-GFP EVs based on the interference (reduction) of the fluorescence signal expected from HER2-DDK:anti-HER2 antibody interaction. Indeed, 1–2*10^8^ antiHER2 EVs remarkably reduced the specific HER2-DDK:anti-HER2 antibody interaction (p value < 0.0001), confirming that near 20% of secreted EVs were exposing a functional moiety that, despite the presence of GFP, retained a measurable binding activity and competition with a canonical anti-HER2 antibody (Fig. 3C). We also obtained the same qualitative indication from immunoprecipitation (IP) experiments using HER2-DDK or anti-GFP antibody to capture the fusion proteins on EVs. HER2-DDK/anti-FLAG or anti-GFP/Protein G beads were incubated with EV-containing media diluted according to the relative particle concentration detected by imaging flow cytometry. IP-GFP bands at the corresponding size and SYNTENIN bands confirmed the presence of secreted EVs exposing the recombinant proteins with accessible GFP and Trastuzumab light chain moieties (Fig. 3D). The canonical Trastuzumab was also included in these experiments as a fourth ligand to investigate competition effects. Notably, antiHER2 EVs, but not CD81-GFP EVs, reproducibly competed with Trastuzumab, reducing the IP signal by about 50% (Fig. 3E). Although we cannot exclude a sub-optimal interaction due to antibody-mediated aggregation and the presence of GFP, antiHER2 EVs were capable of specific interactions with a human recombinant HER2 receptor. These results encouraged the use of these GFP EV populations (now referred to as “recombinant EVs”) for EV-cell interaction studies.

### Recombinant EVs show heterogeneous interactions with breast cancer cells

**Fig. 4:**
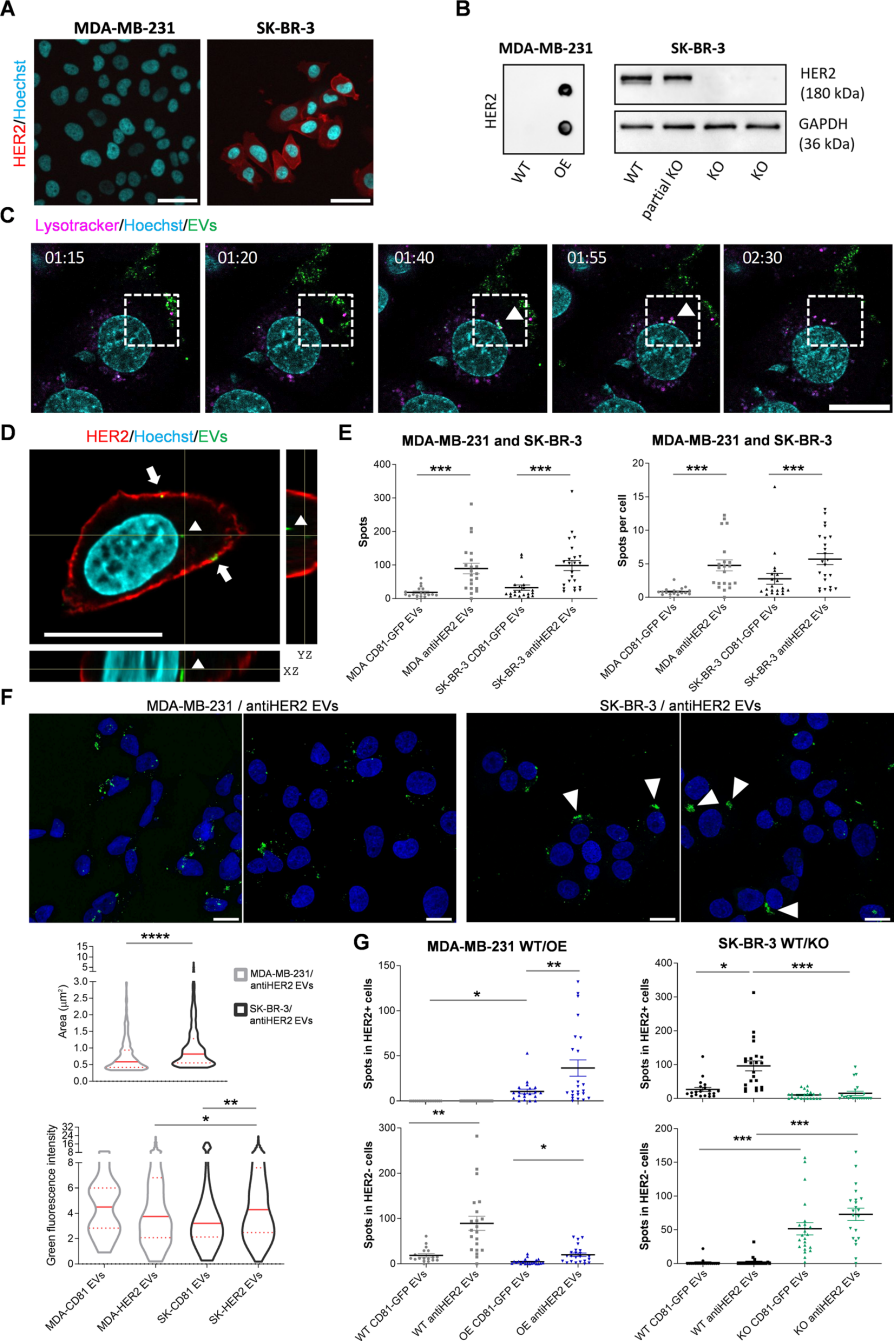
Recombinant EVs show heterogeneous interactions with breast cancer cells. **A** Immunofluorescence of MDA-MB-231 and SK-BR-3 breast cancer cell lines as HER2 negative or positive cells, respectively. HER2 receptor is in red (Alexa Fluor 633), nuclei are shown in cyan (Hoechst). The indicated scale bar is 50 μm. **B** Left: HER2 protein detection by Dot blot in lysates from wild-type or transfected (OE) MDA-MB-231 cells. Right: Immunoblot for checking the selection of SK-BR-3 cells with HER abrogation (*ERBB2*-KO, or KO). **C** Representative confocal time lapse of recombinant EVs incubated with live cells (time points are indicated). GFP-EVs are shown in green, lysosomes are shown in magenta (Lysotracker red), and nuclei in cyan (Hoechst). The white squares highlight the co-localization between EVs and lysosomes (white arrowhead). The indicated scale bar is 20 μm and the time points expressed as hh:mm. **D** Representative confocal image of fixed SK-BR-3 cells recognizing HER2 (Alexa Fluor 633) and nuclei (Hoechst) after 4 h incubation with CD81-GFP EVs (green spots). XY, YZ and XZ orthogonal views are reported. White arrows or the arrowhead indicate different localization of EVs upon cell interaction. Scale bar is 20 μm. **E–G** Quantification of recombinant EVs in recipient breast cancer cell lines. Cells were incubated with EVs for 4 h, then washed with PBS before fixation and HER2 immunofluorescence. Fourteen Z-stacks were acquired within around 11 μm of total Z-size and the Maximum Intensity Projections have been analyzed with an automated pipeline (using CellProfilerTM 4.0.7). Graphs report Mean and SD of the spot distribution from three independent experiments (*P < 0.05, **P < 0.01, ***P < 0.001, ****P < 0.0001). E) GFP + spots per field (left) or per cell (right) are quantified for CD81-GFP or antiHER2 EVs on MDA-MB-231 and SK-BR-3 WT. F) Representative images of the analyzed confocal acquisitions. Accumulation of GFP + spots in SK-BR-3 cells is indicated with arrowheads. Nuclei are in blue and the scale bar is 20 μm. Bottom graphs report GFP + spots’ area and fluorescence intensity. G) GFP + spots are quantified for CD81-GFP or antiHER2 EVs on MDA-MB-231 (WT and HER2 OE) and SK-BR-3 (WT and KO), with distinction of HER2 + (top) and HER2- (bottom) cells based on HER2 IF

By confocal and live cell imaging, we studied the in vitro tropism of recombinant EVs on different breast cancer cells. As popular cellular models for differential HER2 expression, we used SK-BR-3 and MDA-MB-231 breast cancer cell lines and confirmed the respective positive or negative receptor expression by immunofluorescence and immunoblotting (Fig. 4A and Fig. S3A). Next, to better investigate the biological relevance of the receptor on potential EV-cell interactions, we established reciprocal isogenic cells by ectopically expressing HER2 (MDA-MB-231 OE) or abrogating HER2 by CRISPR/Cas9-mediated knock-out (SK-BR-3 KO) (Fig. 4B and Fig. S3B). In parental cells (WT), recombinant EVs were tracked for up to ten hours of incubation with living cells grown under standard conditions. A ratio of about 30,000 bulk EVs per seeded cell (1.5*10^8^ bulk EVs) was maintained to ensure GFP detection (Fig. S3C). Time-lapse confocal imaging (Fig. 4C) showed EV-cell interaction and internalization followed by partial lysosome accumulation, as demonstrated by some LysoTracker co-localizing spots. These results confirm that heterologous EVs could undergo different fates upon endocytosis, including lysosomal degradation or recycling [48].

In parallel, cells were incubated with an equivalent amount of GFP-positive EVs for four hours before washing, fixation, and imaging. We performed automated confocal imaging analysis on Z-stack maximum intensity projection by quantifying GFP spots in a single cell area defined by the HER2 receptor as shown in Fig. 4D. The exposure of both MDA-MB-231 and SK-BR-3 resulted in an equivalent cumulative number of GFP spots respectively detected in the CD81-GFP and antiHER2 conditions. In these settings and in both cell lines, antiHER2 EVs were more prone to interact compared to CD81-GFP EVs (Fig. 4E left). Interestingly, this trend remained when normalizing the number of heterogeneous spots against the number of EV-receiving cells, indicating a promiscuous EV-cell interactome generally shown by control and antiHER2 recombinant EVs (Fig. 4E right). Analyzing the fluorescence distribution more in detail, we observed differential mean spot areas among the two cells lines, with antiHER2 EVs contributing to 0.4 μm^2^ on average increase in SK-BR-3 compared to MDA-MB-231 cells (see arrows in the images and spot area quantification in Fig. 4F). Interestingly, this parameter was paralleled by a significant increase in antiHER2 EV signal intensity in SK-BR-3 cells, while no significant changes were observed in MDA-MB-231 cells (Fig. 4F). These data indicated that both cell lines could variably interact with all the exposed EVs, but the presence of the HER2 receptor in SK-BR-3 cells contributed to additional interactions with antiHER2 EVs, resulting in a measurable advantage of antiHER2 EVs for these cells compared to MDA-MB-231 cells. To this view, the number of spots represented a more stringent parameter to assess EV homing in vitro.

To get more insights into the specificity of antiHER2 EVs, we performed the same high-content imaging analysis in MDA-MB-231 and SK-BR-3 cells with ectopic expression or abrogation of the HER2 receptor, respectively (Fig. S4). Interestingly, the tropism of recombinant EVs was significantly influenced by receptor dosage in the cell subpopulations, dictating an accumulation of GFP spots in HER2-positive MDA-MB-231 cells and a dramatically reduced signal in the SK-BR-3 cells with abrogated HER2 receptor (Fig. 4G). In particular, the number of spots of antiHER2 EVs was significantly higher than that of CD81 EVs in MDA-MB-231 OE cells (Fig. 4G top left) with less impact in the fraction of HER2-negative MDA-MB-231 cells (Fig. 4G bottom left). Notably, the cumulative spot distribution in HER2-negative cells was higher in parental cells and coherent because not all cells were transfected or ectopically expressed the receptor at the same level. Consistently with the indication of MDA-MB-231 cells, the number of detected spots of antiHER2 EVs in SK-BR-3 KO was severely affected by the absence of HER2 receptor (Fig. 4G top right), even losing the original difference in uptake with respect to CD81 EVs (Fig. 4G bottom right). Overall, these results suggest the existence of multiple mechanisms of EV-cell interactions with potential cell lineage-dependent dynamics partially contributed by individual cell surface receptors. From this perspective, imaging tools can be exploited to study EV-cell recruiting mechanisms in vitro to select and enrich EVs with improved functionalization properties.

### HER2 receptor exposure influences the sensitivity to heterologous doxorubicin-EVs

**Fig. 5:**
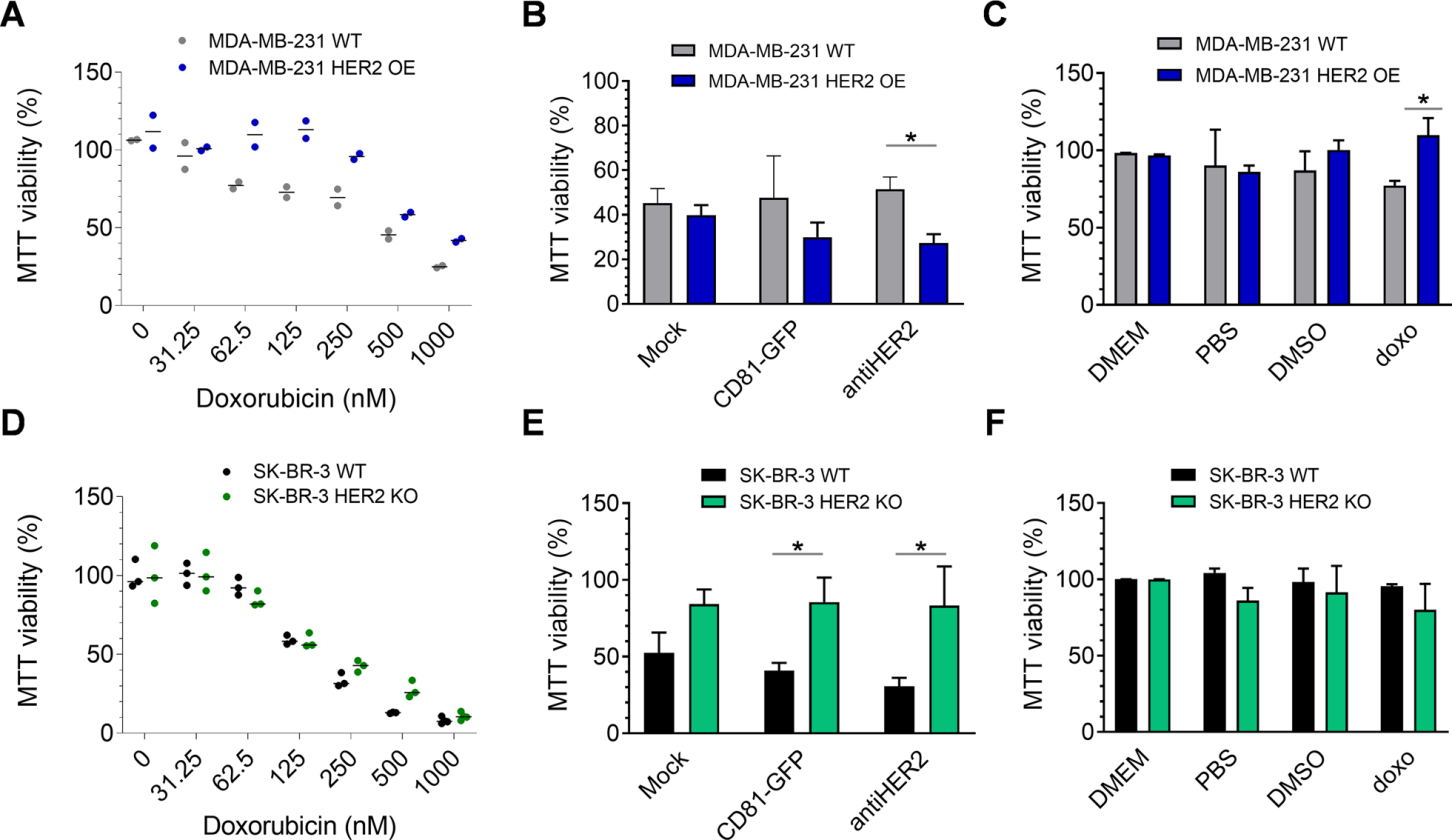
Functional interactions of recombinant doxo-EVs with breast cancer cells. **A, D** Cell viability following doxorubicin treatment of MDA-MB-231 WT and HER2 OE (**A**) and SK-BR-3 WT and KO (**D**) for 72 h. **B, E** MTT assay on MDA-MB-231 WT and HER2 OE (**B**) and SK-BR-3 WT and KO (**E**) after 72 h of incubation with recombinant doxo-EVs (60 nM of secreted doxorubicin). Effect of doxo-EVs was normalized on recombinant EVs without the drug and doxorubicin alone at the corresponding concentration (60 nM). **C, F** MTT controls including doxorubicin alone are reported for the same cells. Graphs show Mean and SD of three biological replicates (*P < 0.05)

To investigate the functional consequences of heterologous EV-cell interactions, we applied an EV pre-isolation strategy to optimize the recovery of vesicles with co-secreted doxorubicin (doxo-EVs) [49, 50]. In this case, we also used pre-isolation drug loading approaches to avoid potential interference on the recombinant EV interactome previously characterized by high-content imaging. The main steps we followed included doxorubicin treatment of transfected HEK293T cells with different doses and timing, to then proceed through particle sedimentation by DUC and mass spectrometry. We tested three drug concentrations (0.5, 5, and 15 µM) along with treatment (3 or 9 h) and release-kinetic schedules (6, 20, or 24 h). According to results shown in Fig. S5A, we selected ten µM for 3 h of treatment and 24 h of release as an acceptable protocol for preserving cell viability, doxorubicin abundance, and comparable NTA profiles. The relative concentration of doxorubicin per particle was determined through a calibration curve (Fig. S5B) run in parallel with NTA analyses (Fig. S5C). The number of recovered particles correlated with doxorubicin concentration in the range of 0.3 to 4 µg per 10^12^ particles (Fig. S5D). To understand whether the doxorucibin cargo of recombinant EVs could induce predictable effects according to the observed cell-interaction behavior, we performed viability assays in MDA-MB-231 cells expressing or not the HER2 receptor. Increasing concentrations of doxorubicin alone caused different cytotoxic effects in these cells, becoming more resistant to the treatment upon HER2-overexpression (IC_50_ of 158 nM for WT and 346 nM for OE condition). This outcome was probably related to the phenotypic advantage conferred by the receptor [51] or such indirect effects on drug diffusion (Fig. 5A). Then, we performed MTT assays by treating cells with secreted doxo-EVs for 72 h with a retrieved drug concentration of 60 nM. We included Mock-doxo EVs and free drugs (doxorubicin and DMSO) as controls. Interestingly, doxo EVs were generally more effective that the free drug, confirming previously reported data [50]. Remarkably, antiHER2 EVs elicited more cell killing activity in HER2-expressing cells compared to parental ones (Fig. 5B), coherently with the contribution of a receptor-mediated EV internalization observed in imaging experiments. The altered viability can be magnified if considering the different sensitivity of the two cell populations to doxorubicin alone (Fig. 5C), confirming the functional specificity of antiHER2 EVs compared to control EVs and the cytotoxic impact of EVs alone. On the other hand, given their stable growth after antibiotic selection and differential behavior with recombinant EVs, we performed viability assays using WT and HER2-KO SK-BR-3 cells. In this case, treatment with free doxorubicin caused similar cytotoxic effects, with a calculated IC_50_ of 144 and 147 nM, respectively (Fig. 5D). In line with the observed EV-cell interactome, antiHER2-doxo EVs showed slightly better efficacy than CD81-GFP-doxo EVs but were far less effective in HER2 KO cells, reverting the trend observed in MDA-MB-231 OE cells (Fig. 5E). These cells had a 10% cell viability reduction with the free drug, while doxo-EVs killed almost 50–65% of wild-type cells, demonstrating measurable effects of exposed EVs (Fig. 5F). The dramatic opposite effect of antiHER2 EVs in the two different cell lines indicated that exposed receptors could functionally influence the interaction spectrum of heterologous EVs with subsequent, predictable cargo internalization/bioavailability. Summarizing the quantification of antiHER2 EVs, AlphaLISA, and imaging experiments, transfected HEK293T released in 48 h a 20% functionalized EV fraction eliciting HER2 receptor binding and selective drug delivery.

### Recombinant EVs reach breast orthotopic tumors and share their full-length mRNA cargo

**Fig. 6:**
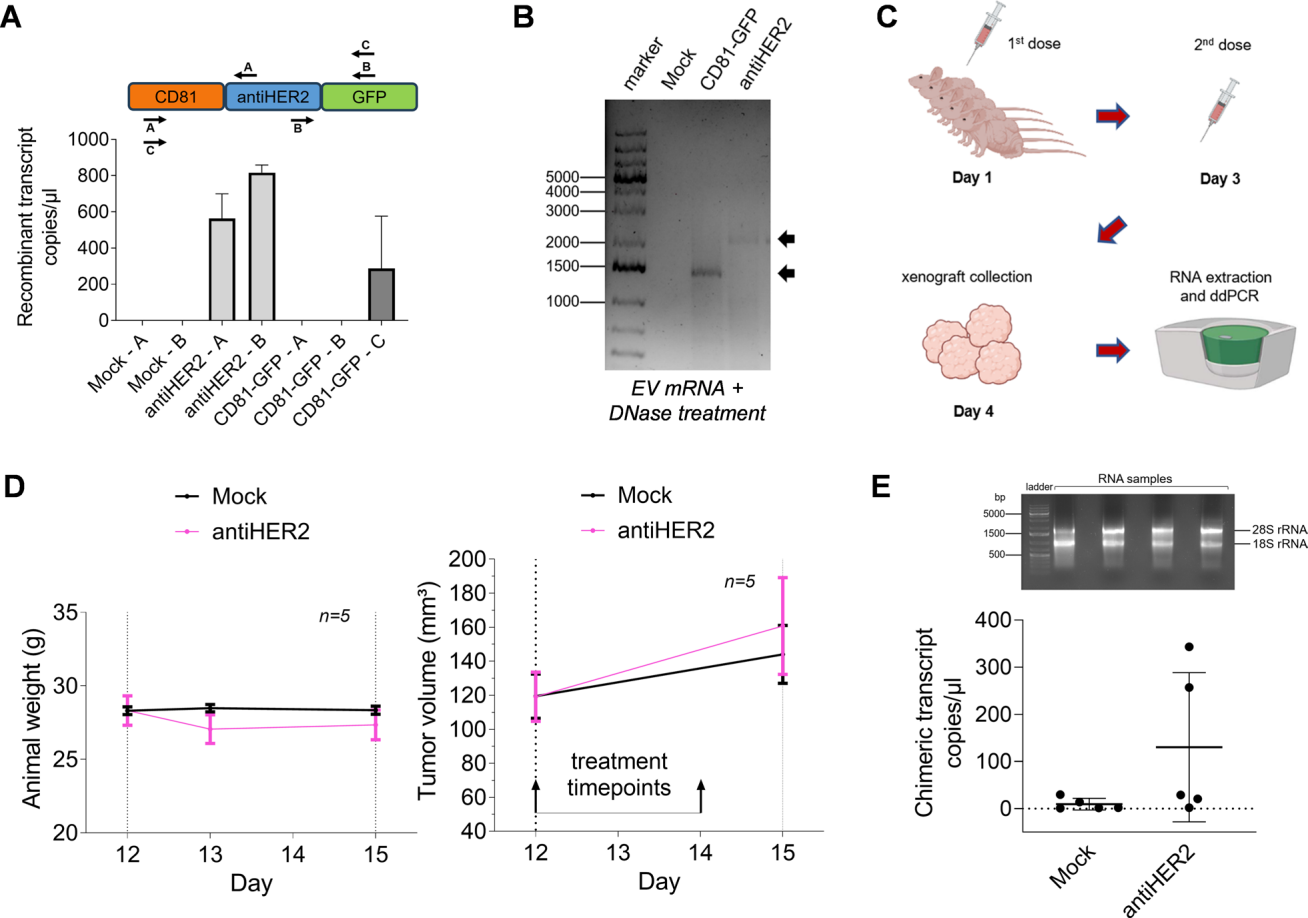
Recombinant EVs reach breast orthotopic tumors and share their full-length mRNA cargo. **A** Schematic representation of primers tested in ddPCR to detect the recombinant transcripts in EV-RNAs. The histogram below shows the observed transcript copy number per microliter in ddPCR experiments. Mean and SD refer to two independent experiments.** B** cDNA synthetized from EV-RNA was amplified for detecting the recombinant transcripts in the full-length form (indicated by arrows) on agarose gel. **C** Schematic representation of the in vivo study, from mice treatment (5 per condition) to recombinant EV-RNA detection by ddPCR in tumor xenografts. Mock doxo-EVs and antiHER2 doxo-EVs were tested. Subcutaneously injection was performed with a doxorubicin concentration of 0.5 µg/kg at tumor implantation site on day 0 and day 2. **D** Animal weight measured during the in vivo study at day 12, 13 and 15 (left) and tumor volume measured before and after mice treatment, at day 12 and 15, respectively (right). Mean and SEM are reported in the graphs. **E** Representative agarose gel of the RNA quality (18S and 28S rRNAs, 2 μg RNA/well) obtained from tumor xenografts (top) and copies/µl of recombinant transcripts detected in tumor xenografts (bottom). Each dot corresponds to one animal

Since the EV-mediated communication results in recipient cell phenotypic changes and some of these changes are related to RNA biology, heterologous EVs represent an opportunity to transfer high-molecular weight RNA payloads, while detecting cargo molecules potentially escaping degradation in recipient cells [52]. We therefore prepared doxo-EVs from HEK293T cells for an in vivo study to detect horizontal mRNA delivery. The vesicular RNA cargo represents one of the best sources to be detected when combined with sensitive technologies such as digital droplet PCR (ddPCR) [53, 54]. To understand whether recombinant EVs harbored the corresponding mRNAs, we designed a ddPCR assay to specifically detect relevant transcript fragments, which were indeed amplified with different primer sets (Fig. 6A). Interestingly, the whole chimeric sequences encoding the fusion proteins were amplified by PCR from the EV-RNA and showed the presence of the full-length amplicons also on agarose gel (Fig. 6B). We then moved to the in vivo study involving female athymic nude mice bearing JIMT-1 human breast tumor xenografts. JIMT-1 cells are known to be refractory to HER2 blockade and trastuzumab-resistant [55] [56] and represented a first choice to avoid HER2-mediated cytotoxicity preventing the detection of horizontally transferred molecules, including recombinant RNA and doxorubicin at the low working concentration we obtained. Mice were injected twice with doxo-EVs at the same doxorubicin concentration (0.5 µg/kg), on day one and day three, to be then sacrificed on day four (Fig. 6C). As expected from the relative low dosage of doxorubicin administered, no statistically significant variations were observed in animal weight and tumor volumes among the different treatments (Fig. 6D). However, we analyzed the RNA content of *ex-vivo* tumor cells to verify a detectable cargo of recombinant EVs. We isolated total RNA from tumor cell xenografts and used this template to detect EV-derived fusion protein-encoding transcripts by ddPCR. Interestingly, antiHER2 fusion transcripts were detected in tumor xenografts from at least 2 out of 5 animals, demonstrating a direct transfer of > 2 KB recombinant mRNA from heterologous EVs in vivo (Fig. 6E).

## Discussion

Several studies have reported tetraspanins as valuable tools for engineering and tracing cell-secreted EVs with fluorescent markers [44, 57, 58]. Specifically, CD81 was fused with fluorescent reporters [59, 60] or used as a direct targeting moiety against human placental laminin [61]. In this work, CD81 fusion proteins were exploited to track and quantify the fraction of recombinant EVs over the secreted vesicle sub-populations. The over-expression of fusion proteins was well-tolerated by HEK293T cells and, interestingly, the intracellular expression levels strongly correlated with the relative abundance of EVs containing the protein of interest. This observation is relevant for predicting and optimizing the secretion of heterologous, functionalized EVs. Notably, as mainly indicated by imaging flow cytometry in the case of CD81-GFP EVs, the bulk of detected fusion proteins was not 100% vesicular, therefore fusion proteins could be subjected to parallel routes of packaging, clustering, and secretion [62]. Nevertheless, the ectopic CD81 expression stimulated EV secretion and slightly influenced the size of recovered particles. Cryo-EM experiments confirmed this trend but also presented multi-layered vesicles. Multi-lamellarity was circumstantially described but not yet elucidated if it derives from sample isolation procedures or represents discrete formations with potential biological roles [63, 64]. Since this parameter changed across the tested conditions and mainly characterized the Mock condition (~ 30% of bulk EVs), we reasoned that this parameter could be influenced by the relative single-EV protein content and/or turnover of endo/exocytosis processes. Further research involving dedicated strategies of single EV analysis, perhaps including tomography, is needed to address this aspect.

Seminal studies have already addressed the orientation of the extracellular domain of tetraspanins, including CD81, on the plasma membrane, showing that exposed sub-regions can have different degrees of conservation and serve homo or hetero interactions with tetraspanins [65]. Proteins like CD63, CD9, and CD81 have been recently proposed as a scaffold for inner and outer membranes to display fluorescent reporters, assuming a cell-membrane-equivalent protein orientation. There is circumstantial evidence that SCAMP3 and CD9 transmembrane proteins might configure a reversed topology on EVs compared to the plasma membrane [66, 67], and this outcome could be influenced by a repeated release turnover or budding *versus* fusion processes [68], potentially generating mixed orientations. By competitive interaction and imaging assays, we demonstrated that almost the total fraction of antiHER2 EVs presented an outward topology responsible for the specific binding to the HER2 receptor. In perspective, sorting specific EV sub-populations could allow investigation of the chimeric protein enrichment with a desired topology and in connection with elicited functions in recipient cells.

Despite the presence of GFP virtually contributing to a sub-optimal HER2 receptor binding, we had a chance to monitor EV-cell interactions in vitro. The mechanism underlying the preferential recruitment of heterologous EVs is not well understood. The proteo-lipid composition on cell and EV membranes may influence EV internalization and the propensity to uptake EVs [69]. In our EV-cell imaging experiments, a comparison of two distinct breast cancer cell lines, i.e. the HER2-positive SK-BR-3 and the HER2-negative MDA-MB-231 cells, showed the same cumulative number of GFP spots upon exposure to the different recombinant EVs for a few hours. Therefore, different interactions could result from intrinsic cell properties affecting the EV distribution in vitro, as demonstrated by the general detection of control CD81-GFP EVs. In these conditions, the slightly higher green fluorescence intensity and spot areas were the only parameters informing on the specificity of antiHER2 EVs. However, when we performed the same experiments in isogenic cells manipulated for the expression/exposure of HER2 receptor we observed a remarkable positive engagement of recombinant EVs in HER2-positive cell populations dictated by Trastuzumab light chains in our fusion protein and contributing a 20% of functionalized antiHER2 EVs upon 48 h transfection of HEK293T cells. These experiments indirectly demonstrate that EV-cell interactions are contributed by different mechanisms (possibly involving either proteins, lipids, or eventually nucleic acids) that ultimately reduce the fraction of valuable EVs, as also discussed by Limoni et al. [27] indicating that a high concentration of particles was crucial to detect a selective targeting of HER2-positive SK-BR-3 cells. From this study, we reasonably concluded that the number of EV spots is a useful readout to predict and potentially optimize the EV-cell interaction behavior with different cell populations without involving abnormal EV density exposures. A comprehensive characterization of the surfaceome [70, 71], possibly including glycans [72], of a desired cell population could help customize a multi-modal targeting with enhanced specificity.

Once interacting during the time frame analyzed, live imaging acquisitions indicated a partial co-localization of green spots with lysosomes, suggesting that EVs may have different trafficking rates or follow non-degradative routes within recipient cells [48, 73]. Albeit the characterization of drug encapsulation efficiency and release kinetics need to be implemented and possibly controlled overtime, our results confirm the superior cytotoxic effects of secreted doxorubicin-EVs on HER2-positive breast cancer cells compared to the free drug, as already observed in other cell types [50]. This aspect would represent an advantage in vivo for reducing the effective drug dose and limiting off-targets. In line with cell-interaction assays, the cell-killing activity of recombinant doxorubicin-EVs was significantly mitigated upon removing the cell surface receptor, suggesting that a significant fraction of EVs can have a predictable outcome upon internalization. Therefore, since cell viability experiments in parental and HER2-manipulated breast cancer cells functionally confirmed the differential EV-cell engagement, we reasonably concluded that the characterization of cell-interaction spectrum is a priority to optimize heterologous EVs as clinically suitable tools for targeted delivery.

Finally, using trastuzumab-resistant human breast cancer cells for a mouse xenograft study, we showed that a > 2 KB recombinant mRNA could be horizontally shared in vivo and sensitively detected by ddPCR assays. This approach could be applied to quantify recombinant RNA in tissue sections and address the tumor penetration capacity of heterologous EVs, potentially delivering a controlled RNA payload to selected tumor cells.

## Conclusions

In conclusion, we provide experimental evidence that CD81 fusion proteins are well-tolerated by HEK293T cells and accumulate on secreted EVs according to intracellular expression levels. Our results show that EV-cell interactions are dictated by multiple mechanisms underlying uptake dynamics and internalization that individual cell receptors could marginally contribute; therefore, specific EV-cell interaction studies are required to improve the efficiency of 20% of functionalized EVs over the bulk-secreted vesicles. This study describes imaging experiments as a valuable readout to characterize and enhance HER2-targeting properties of functionalized EVs predicting selective cell-killing activities with a simultaneous drug payload. We also discuss the profiling of cellular surfaceomes to comprehend EV-cell interactions better, leading to a more efficient design and purification of EV-based nanovehicles. In addition, we provided evidence of high-molecular-weight recombinant mRNA horizontal sharing in vivo by heterologous EVs, proposing new pipelines to study the penetrability of transferred cargo in tumor cells and prospecting comparative studies with other lipid/hybrid formulations to advance our portfolio of targeted delivery systems.

## Supplementary Information

Additional file 1: Fig. S1. Expression of CD81 fusion proteins in viable HEK293T cells. A Recombinant proteins CD81-GFP and CD81-antiHER2-GFP (“antiHER2”) are depicted, with light chains of trastuzumab (antiHER2 moiety, blue) and turboGFP (GFP, green). B Differential expression of CD81-fusion proteins in HEK293T cells after 48 hours of transfection with Lipofectamine 3000. Same amount of plasmid DNAs was used for each condition and images were acquired using a Spinning Disc confocal microscope keeping constant all the acquisition parameters. Mock condition is reported as technical control, while cytosolic EGFP as a biological comparison for intracellular localization (pEGFP-N1, Addgene). Scale bar: 50 μm.

Additional file 2: Fig. S2. Direct and competitive AlphaLISA assays. A Sandwich designed for AlphaLISA assay to detect the binding of in vitro-translated antiHER2 protein to recombinant HER2 (DDK-/FLAG-tagged). Alpha Count fold change shows binding specificity at dilution 1 to 40 of antiHER2 protein in three independent experiments. B AlphaLISA competitive assay validation (see also Fig. 3D). As shown in the graph, high Alpha Counts were obtained only in the presence of all the sandwich components.

Additional file 3: Fig. S3. HER2 expression and EV detection optimization for confocal acquisitions. A, B Validation of breast cancer cell lines for EV interaction experiments. Characterization of MDA-MB-231 and SK-BR-3 cells for HER2 expression by WB (A). IF confocal images show abrogation of HER2 expression in SK-BR-3 KO cells. Scale bar: 50 μm (B). C Mock EVs were tested as negative control compared to GFP-positive EVs for EV uptake acquisitions. GFP-EVs are shown in green, lysosomes in magenta (Lysotracker red), and nuclei in cyan (Hoechst). Scale bar: 10 μm.

Additional file 4: Fig. S4. HER2 expression manipulation in isogenic breast cancer cells. Representative confocal image of recipient cells fixed after 4 hr incubation with CD81-GFP or antiHER2 EVs (green spots). HER2 is detected by IF (Alexa Fluor 633) and nuclei are stained with Hoechst. Scale bar: 20 μm.

Additional file 5: Fig. S5. Doxo-EV profiling. A Different doxorubicin concentration (5, 10 or 15 μM) and incubation time (3 or 9 hr) on transfected HEK293T cells were tested, combined with variable EV release timing (6, 20 or 24 hr), in order to determine the optimal conditions for doxo-EV generation. Y axis reports doxorubicin concentration retrieved from isolated EVs by UHPLC-MS (see “Methods”). B Standard curve of known doxorubicin concentrations used to quantify doxorubicin in doxo-EVs by UHPLC-MS (see “Methods”). C NTA analysis of doxo-EVs (mode and mean diameters, particle concentration), similarly to Fig. 2B. D Correlation between doxorubicin concentration retrieved from doxo-EV samples at UHPLC-MS and particle concentration from NTA measurements.

## Data Availability

All data analyzed during this study are included in this published article and supplementary material.

## Abbreviations

ALPHA: Amplified Luminescence Proximity Homogeneous Assay
CMDR: Cell mask deep red
CRISPR: Clustered regularly interspaced short palindromic repeats
Cryo-EM: Cryogenic electron microscopy
ddPCR: Droplet digital PCR
DMEM: Dulbecco's Modified Eagle Medium
DMSO: Dimethyl sulfoxide
DTT: Dithiothreitol
DUC: Differential ultracentrifugation
ESCRT: Endosomal sorting complex required for transport
EVs: Extracellular vesicles
HER2: Human Epidermal growth factor Receptor 2
IP: Immunoprecipitation
KO: Knock-out
LC–MS: Liquid chromatography-mass spectrometry
MTT: Dimethylthiazolyl-diphenyl-tetrazolium bromide
MVBs: Multivesicular bodies
NTA: Nanoparticle tracking analysis
OE: Over-expressed
PBS: Phosphate buffered saline
PDGFR: Platelet-derived growth factor receptor
PEI: Polyethylenimine
RT: Room temperature
SDS-PAGE: Sodium dodecyl sulphate–Polyacrylamide gel electrophoresis
tGFP: Turbo Green fluorescent protein
UHPLC: Ultra High Performance Liquid Chromatography
WT: Wild type
