# Supplementary figures and images for: CD81-guided heterologous EVs present heterogeneous interactions with breast cancer cells
Source: J Biomed Sci. 2024 Oct 15;31:92. doi: 10.1186/s12929-024-01084-9 (PMC11475557; doi:10.1186/s12929-024-01084-9)

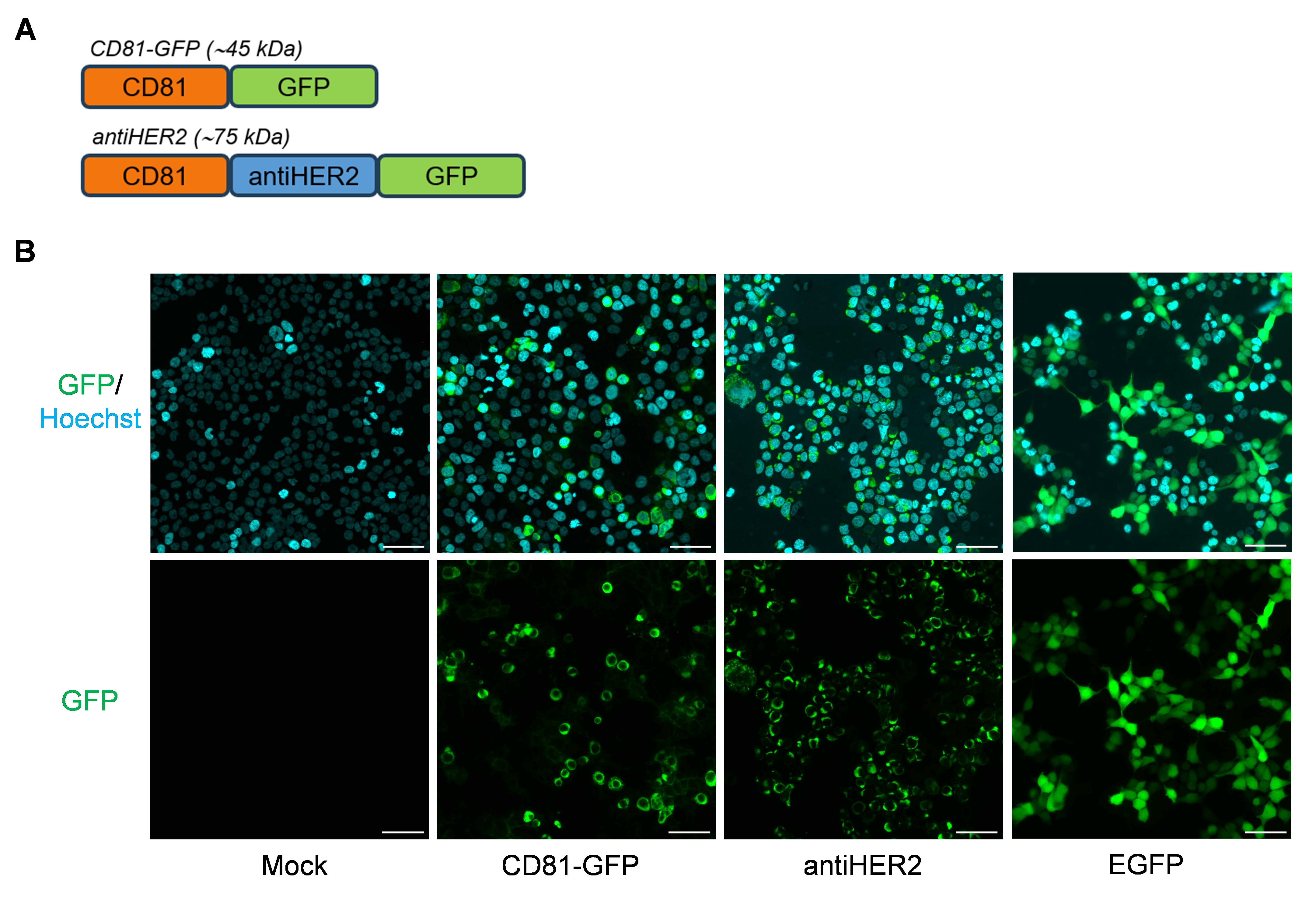

Supplement: Supplementary file 1 — Additional file 1: Fig. S1. Expression of CD81 fusion proteins in viable HEK293T cells. A Recombinant proteins CD81-GFP and CD81-antiHER2-GFP (“antiHER2”) are depicted, with light chains of trastuzumab (antiHER2 moiety, blue) and turboGFP (GFP, green). B Differential expression of CD81-fusion proteins in HEK293T cells after 48 hours of transfection with Lipofectamine 3000. Same amount of plasmid DNAs was used for each condition and images were acquired using a Spinning Disc confocal microscope keeping constant all the acquisition parameters. Mock condition is reported as technical control, while cytosolic EGFP as a biological comparison for intracellular localization (pEGFP-N1, Addgene). Scale bar: 50 μm. [file 12929_2024_1084_MOESM1_ESM.tiff]

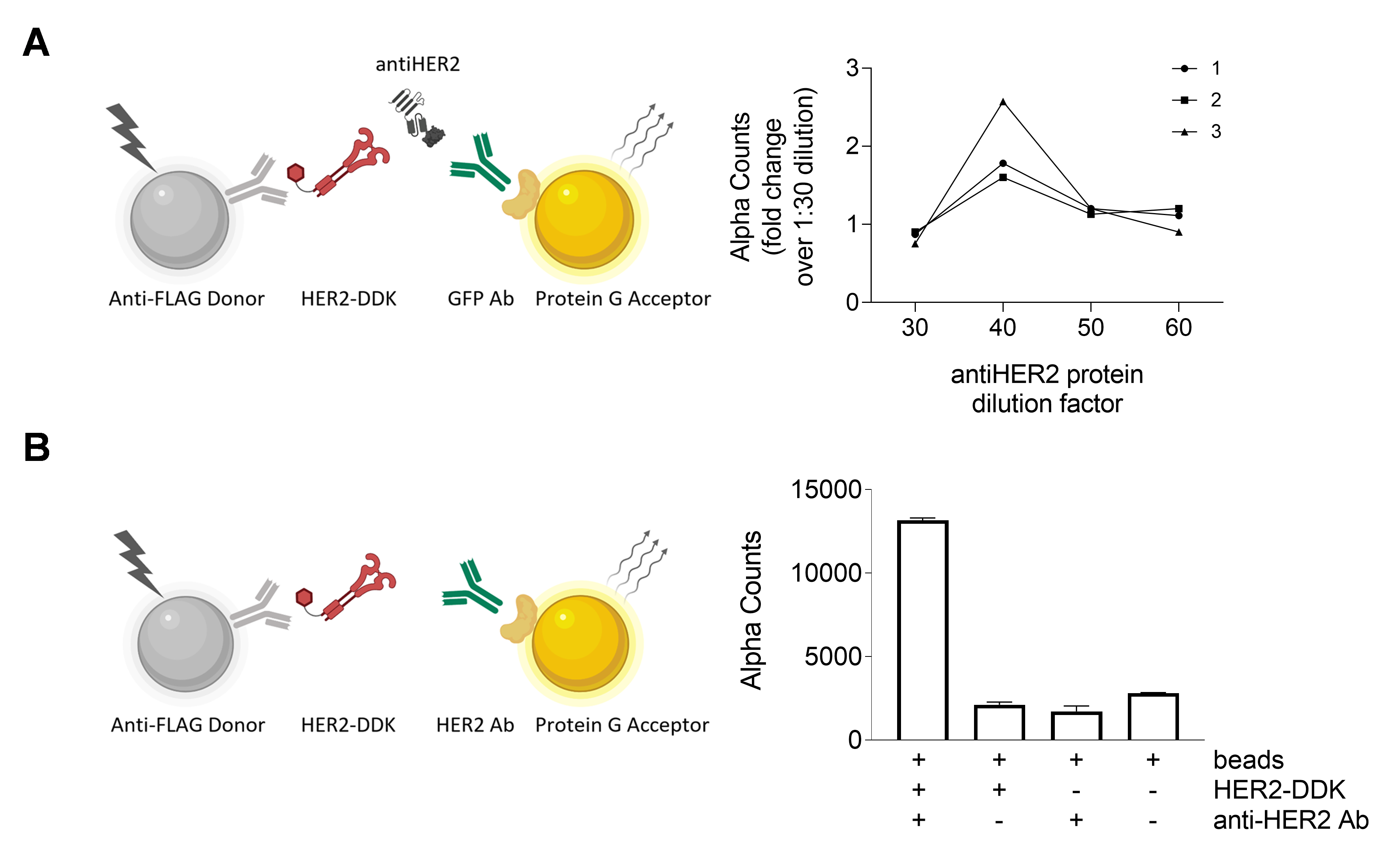

Supplement: Supplementary file 2 — Additional file 2: Fig. S2. Direct and competitive AlphaLISA assays. A Sandwich designed for AlphaLISA assay to detect the binding of in vitro-translated antiHER2 protein to recombinant HER2 (DDK-/FLAG-tagged). Alpha Count fold change shows binding specificity at dilution 1 to 40 of antiHER2 protein in three independent experiments. B AlphaLISA competitive assay validation (see also Fig. 3D). As shown in the graph, high Alpha Counts were obtained only in the presence of all the sandwich components. [file 12929_2024_1084_MOESM2_ESM.tiff]

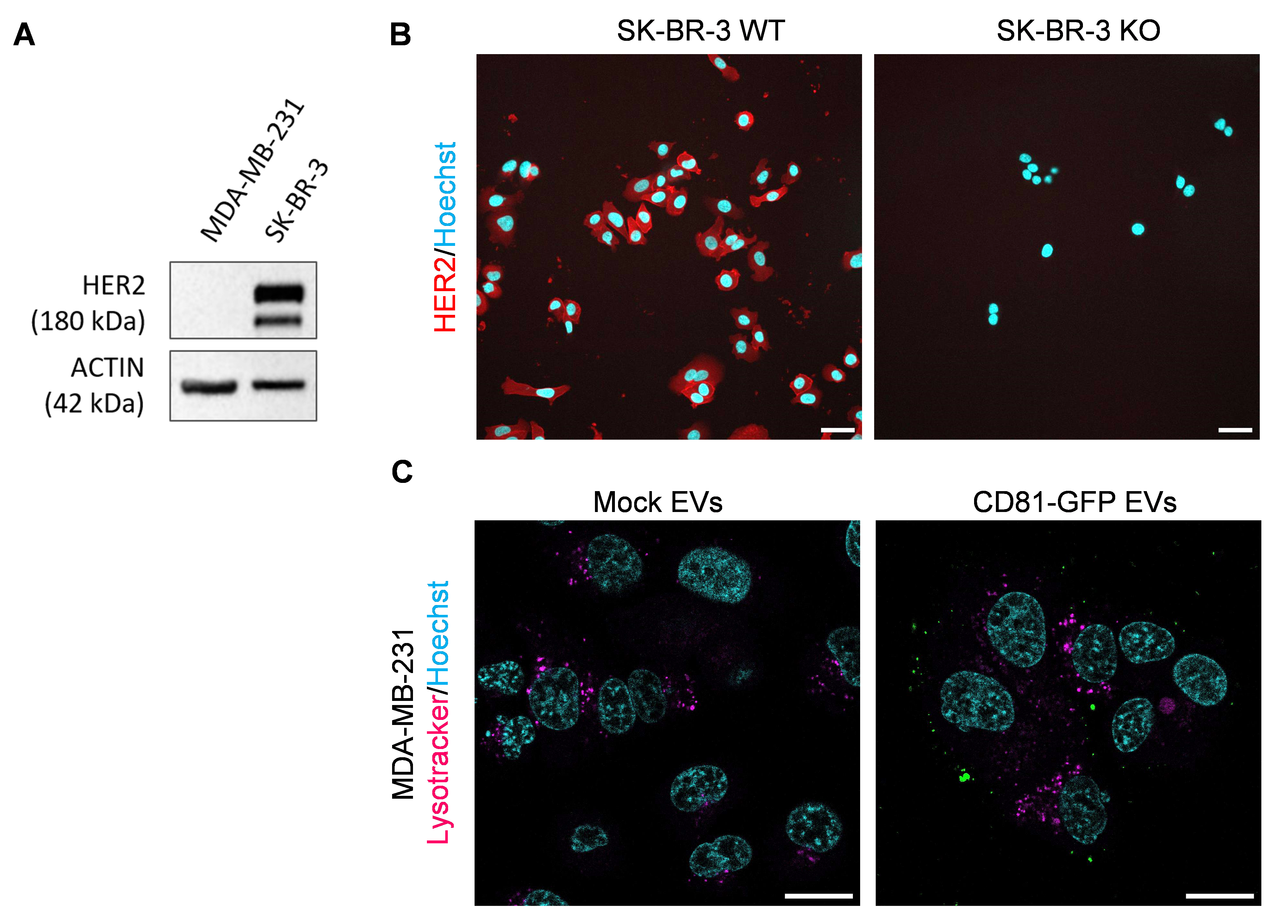

Supplement: Supplementary file 3 — Additional file 3: Fig. S3. HER2 expression and EV detection optimization for confocal acquisitions. A, B Validation of breast cancer cell lines for EV interaction experiments. Characterization of MDA-MB-231 and SK-BR-3 cells for HER2 expression by WB (A). IF confocal images show abrogation of HER2 expression in SK-BR-3 KO cells. Scale bar: 50 μm (B). C Mock EVs were tested as negative control compared to GFP-positive EVs for EV uptake acquisitions. GFP-EVs are shown in green, lysosomes in magenta (Lysotracker red), and nuclei in cyan (Hoechst). Scale bar: 10 μm. [file 12929_2024_1084_MOESM3_ESM.tif]

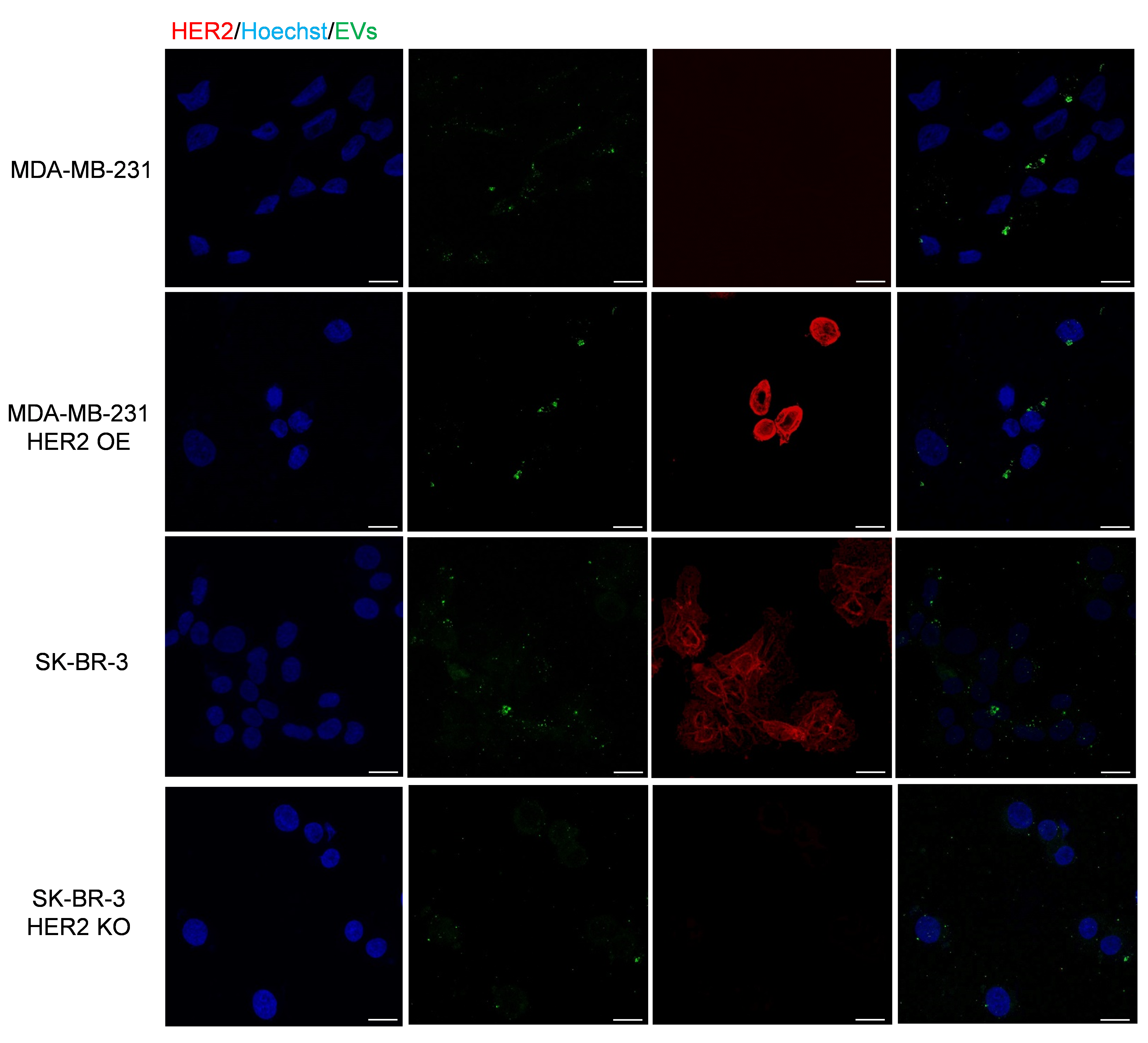

Supplement: Supplementary file 4 — Additional file 4: Fig. S4. HER2 expression manipulation in isogenic breast cancer cells. Representative confocal image of recipient cells fixed after 4 hr incubation with CD81-GFP or antiHER2 EVs (green spots). HER2 is detected by IF (Alexa Fluor 633) and nuclei are stained with Hoechst. Scale bar: 20 μm. [file 12929_2024_1084_MOESM4_ESM.tif]

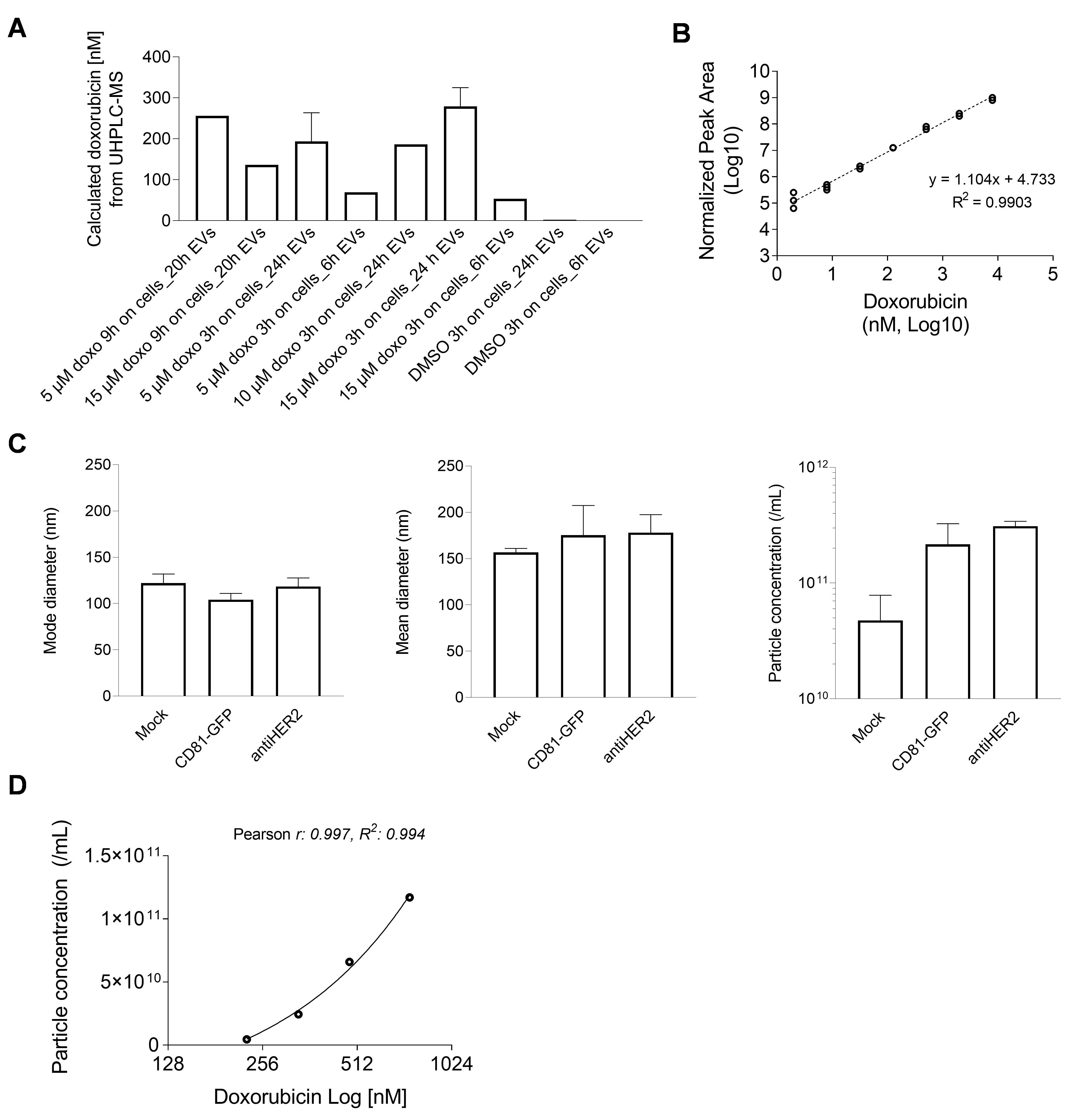

Supplement: Supplementary file 5 — Additional file 5: Fig. S5. Doxo-EV profiling. A Different doxorubicin concentration (5, 10 or 15 μM) and incubation time (3 or 9 hr) on transfected HEK293T cells were tested, combined with variable EV release timing (6, 20 or 24 hr), in order to determine the optimal conditions for doxo-EV generation. Y axis reports doxorubicin concentration retrieved from isolated EVs by UHPLC-MS (see “Methods”). B Standard curve of known doxorubicin concentrations used to quantify doxorubicin in doxo-EVs by UHPLC-MS (see “Methods”). C NTA analysis of doxo-EVs (mode and mean diameters, particle concentration), similarly to Fig. 2B. D Correlation between doxorubicin concentration retrieved from doxo-EV samples at UHPLC-MS and particle concentration from NTA measurements. [file 12929_2024_1084_MOESM5_ESM.tif]
